# Redox‐Dependent Chaperoning of GBF1 Condensates Regulates Seed Germination in Arabidopsis

**DOI:** 10.1002/advs.202520599

**Published:** 2026-05-26

**Authors:** Yunying Wang, Xiaofeng Fang

**Affiliations:** ^1^ Center For Plant Biology School of Life Sciences Tsinghua University Beijing China; ^2^ Tsinghua University‐Peking University Joint Center For Life Sciences Beijing China; ^3^ State Key Laboratory of Green Biomanufacturing Beijing China

**Keywords:** molecular chaperone, phase separation, reactive oxygen species, seed germination, transcriptional condensate

## Abstract

Reactive oxygen species (ROS) are pivotal signals that trigger the transition from seed dormancy to germination, yet the molecular link between ROS and transcriptional regulation has remained unclear. Here, we uncover a redox‐dependent mechanism focusing on the transcription factor G‐box binding factor 1 (GBF1) and its chaperone GBF1‐interacting protein 1 (GIP1). We show that GBF1 undergoes liquid‐liquid phase separation (LLPS) to form condensates that enhance target DNA binding and repress the transcription of the germination‐promoting gene *Cathepsin B‐like protease 3* (*CathB3)*. Both the prion‐like domain and the bZIP dimerization domain are indispensable for GBF1 condensation and DNA binding. GIP1 colocalizes with GBF1 and functions as a molecular chaperone that fine‐tunes condensate size and liquidity, thereby enhancing GBF1's DNA‐binding and repressive capacity. Oxidation of GIP1 by ROS burst during germination abolishes its chaperone activity, leading to aberrant, less dynamic GBF1 condensates and consequent derepression of *CathB3*, which promotes germination. This work identifies a ROS‐sensitive chaperone‐condensate axis as a key molecular gatekeeper of seed germination, revealing how redox signals are translated into developmental decisions through the material properties of transcriptional condensates.

## Graphical abstract

In dormant seeds (low ROS), GBF1 forms liquid condensates to repress the germination gene *CathB3*, and the chaperone GIP1 maintains condensate liquidity and repressive activity. Upon imbibition (high ROS), ROS oxidize GIP1 during germination, impairing its chaperone function. This leads to less dynamic, solid‐like GBF1 condensates and derepression of *CathB3*, thereby permitting germination.

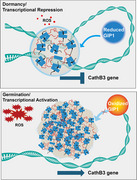

## 1 Introduction

Seed dormancy and germination represent critical developmental checkpoints that determine when a plant initiates its life cycle [1]. The precise timing of this transition is essential for crop establishment and survival under fluctuating environmental conditions [2, 3]. While hormonal networks based on abscisic acid and gibberellin have long been recognized as primary regulators, ROS have emerged as key signaling molecules that promote germination [4, 5, 6]. ROS are known to oxidize mRNAs and hormone‐related proteins to regulate the downstream germination process [7, 8, 9, 10]. Yet, how ROS signals are transmitted to the transcriptional machinery to control gene expression during germination remains poorly understood.

Recent advances have highlighted the role of biomolecular condensation via LLPS in spatiotemporally organizing transcriptional regulation [11, 12]. Transcription factors and co‐regulators often assemble into dynamic condensates that concentrate regulatory complexes and facilitate selective gene expression [13, 14]. Importantly, accumulating evidence indicates that the ROS signal can be transmitted via biomolecular condensation. For instance, yeast ataxin‐2 was proposed to sense the cellular redox state by oxidizing methionine residues within its IDR, thereby inhibiting its condensation [15]. In tomato, the developmentally accumulated ROS in the shoot apical meristem promotes the condensation of TERMINATING FLOWER (TMF), forming transcriptional condensates that repress *ANANTHA* expression to precisely control stem cell fate and flowering time [16]. The material properties of these condensates, such as size, liquidity, and stability, critically influence transcriptional outcomes, with overly solidified assemblies impairing regulatory capacity [17, 18, 19, 20]. Recent work has further demonstrated that heat stress‐induced ROS burst stabilizes the transcriptional condensates of TMF, thereby prolonging vegetative growth [21]. However, whether the material properties of transcriptional condensates deliver ROS signals that control seed dormancy‐to‐germination transitions has remained elusive.

Here, we uncover a redox‐dependent condensate mechanism that fine‐tunes seed germination in *Arabidopsis thaliana*. We identify the transcription factor GBF1 as a regulator that undergoes LLPS to form nuclear condensates, which repress the expression of the germination‐promoting gene *CathB3*. We further demonstrate that GIP1, a previously proposed GBF1‐interacting protein, acts as a molecular chaperone that maintains the liquidity and functional activity of GBF1 condensates. Crucially, ROS oxidize GIP1 and abrogate its chaperone activity, leading to aberrant condensate maturation and derepression of *CathB3*. Together, these findings reveal how a redox‐sensitive chaperone‐condensate axis directly links ROS accumulation to transcriptional control, providing a new paradigm for the molecular regulation of seed germination.

## 2 Results

### 2.1 GBF1 Undergoes Phase Separation In Vitro

**FIGURE 1:**
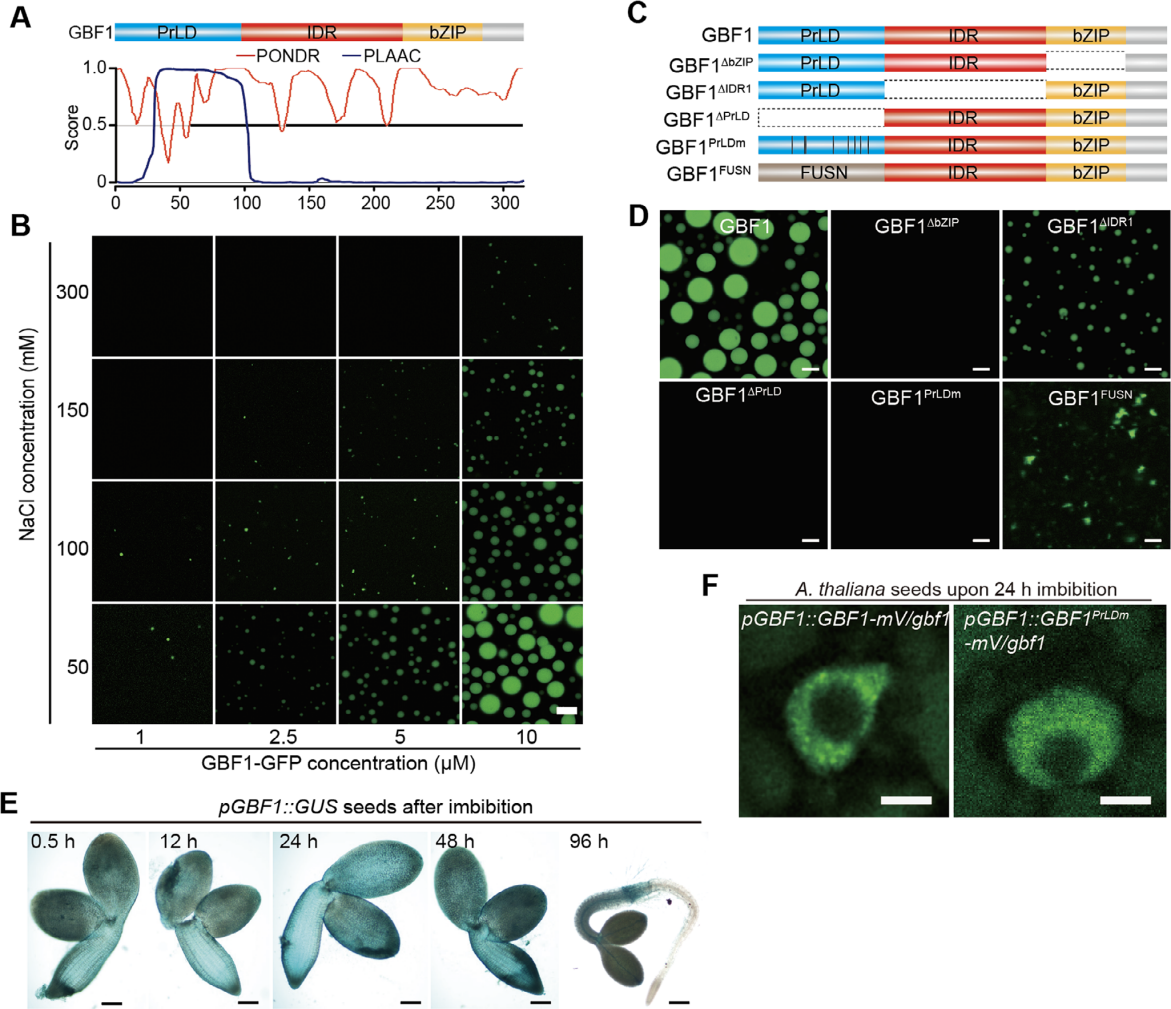
The bZIP transcription factor GBF1 undergoes phase separation in vitro and in vivo. (A) Prediction of the IDR and PrLD of GBF1 by PONDR and PLACC algorithms, respectively. (B) In vitro phase diagram of purified GBF1‐GFP. Scale bar, 10 µM. (C) The protein domain structures of GBF1 and its variants. (D) Representative images of in vitro phase separation for GBF1 and its variants. Scale bars, 5 µM. (E) Spatial expression pattern of GBF1 in imbibed seeds as revealed by histochemical staining. Scale bars, 200 µM. (F) Confocal microscopy of embryos from 24 h‐imbibed seeds of the indicated genotypes. Scale bars, 3 µM.

We have used biotinylated isoxazole (b‐isox)‐mediated precipitation to identify potential phase separation proteins [22]. This approach enabled us to successfully elucidate the function of biomolecular condensates in multiple cellular processes [23, 24, 25, 26]. Among the b‐isox enriched proteins, we found that GBF1, which belongs to the G group of the basic leucine zipper (bZIP) transcription factor family, was highly represented (Figure S1A). Both Alphafold2 and bioinformatic prediction revealed that GBF1 is largely disordered except for a bZIP DNA‐binding domain (Figure 1A; Figure S1B). To verify the ability of GBF1 to undergo phase separation, we purified the GBF1‐GFP in vitro and found that it formed sphere condensates (Figure 1B). GBF1‐GFP condensates were sensitive to ionic strength (Figure 1B). Fluorescence recovery after photobleaching (FRAP) and fusion assays revealed that GBF1‐GFP condensates were dynamic and exhibited liquid‐like behaviour (Figure S1C–E), indicating that GBF1 undergoes LLPS. GBF1 phase separation was unaffected by GFP tag, as untagged GBF1 similarly formed sphere condensates in an ionic strength‐dependent manner (Figure S1F).

Based on prediction, GBF1 was divided into three segments: the N‐terminal prion‐like domain (PrLD), the middle intrinsically disordered region (IDR), and the C‐terminal bZIP domain (Figure 1A). Deletion of the PrLD (GBF1^∆PrLD^) or bZIP (GBF1^ΔbZIP^) abolished GBF1 phase separation in vitro, and deletion of the IDR (GBF1^ΔIDR^) markedly reduced its phase‐separation capacity (Figure 1C,D; Figure S2A). Similar to most other PrLDs [27], the PrLD of GBF1 was enriched with aromatic amino acids, which are often crucial for driving phase separation [28]. Mutation of 11 aromatic residues (phenylalanine and tyrosine) to serine in the PrLD (GBF1^PrLDm^) significantly diminished the phase separation capability of GBF1 (Figure 1C,D, Figure S2A). In contrast, replacing the PrLD with heterologous PrLD from the fused in sarcoma (FUS) [27, 29, 30] largely restored the phase separation of GBF1 (Figure 1C,D; Figure S2A). The bZIP domain is known to form dimers, which are critical for DNA binding [31]. These results indicate that both bZIP dimerization and the multivalent interactions from PrLD are necessary for GBF1 phase separation.

The G group of the bZIP transcription factor also contains GBF2, GBF3, bZIP16, and bZIP68 [31]. Similar to GBF1, bZIP16, and bZIP68 were enriched by b‐isox and contain PrLDs and IDRs (Figure S1A,G,H). Compared to GBF1, bZIP16, or bZIP68 alone exhibited weaker or no phase separation capacity in vitro (Figure S1I,J). However, both bZIP16 and bZIP68 were incorporated into GBF1 condensates and enhanced GBF1 condensation (Figure S1K), suggesting that these three proteins may function redundantly via phase separation. In agreement, it was reported that bZIP16, bZIP68, and GBF1 together regulate nuclear photosynthetic genes during photomorphogenesis [32].

### 2.2 GBF1 Forms Nuclear Condensates In Vivo

To test if GBF1 undergoes phase separation in cells, we first expressed full‐length GBF1 and its variants in yeast cells, which have been used to assess the condensation of plant proteins [22]. Similar to the observations obtained in vitro, full‐length GBF1 and chimeric FUSN‐GBF1 formed nuclear condensates, whereas other variants had either fewer or no condensates (Figure S2B). Expression in tobacco epidermal cells further confirmed this result (Figure S2C). Consistent with in vitro co‐partitioning, bZIP16 and bZIP68 colocalized with GBF1 condensates in tobacco cells (Figure S2D).

*GBF1* was found to be expressed in seed embryos upon germination [33]. Indeed, our histochemical staining of GUS driven by the *GBF1* promoter revealed a strong signal in the embryos of imbibed seeds (Figure 1E). To confirm the condensation of GBF1 in vivo, we generated a complementation line by expressing the full‐length coding sequence (CDS) of GBF1 fused with the CDS of a yellow fluorescent protein under its native promoter (*pGBF1::GBF1‐mVenus*) in *gbf1* knockout plants. Distinct GBF1‐mVenus condensates were observed in the nuclei of the seed embryo (Figure 1F). In contrast, GBF1 with PrLD mutated (*pGBF1::GBF1^PrLDm^‐mVenus*) was diffused homogenously (Figure 1F).

Together, these results indicate that GBF1 undergoes phase separation in vitro and in vivo, and that the aromatic residues in PrLD are crucial for this process.

### 2.3 Phase Separation of GBF1 Promotes Target DNA Binding

**FIGURE 2:**
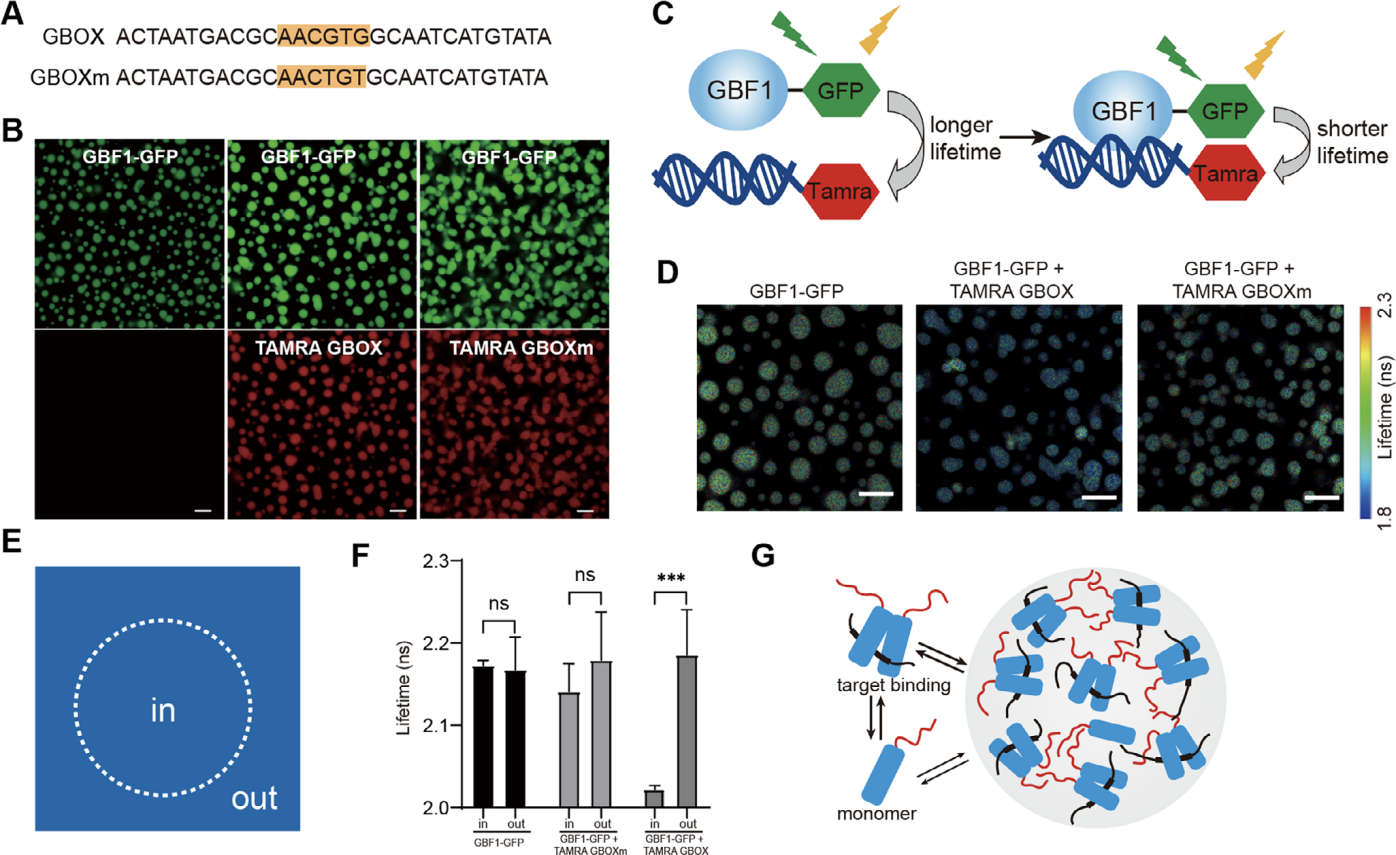
Phase separation of GBF1 enhances its binding to target DNA. (A) Sequences of the 30‐bp DNA probes derived from the *CathB3* promoter. The G‐box core motif is highlighted. *GBOXm* contains a mutated motif. (B) Partitioning of *TAMRA‐GBOX* and *TAMRA‐GBOXm* probes into GBF1‐GFP condensates formed in vitro. 10 µm GBF1‐GFP and 100 nM DNA probe were incubated for 30 min before imaging. (C) Schematic of the FLIM‐FRET assay to measure DNA binding. A shorter fluorescence lifetime of the donor (GFP) indicates FRET occurrence due to binding to the acceptor (TAMRA)‐labeled DNA. (D) Fluorescence lifetime image of a GBF1‐GFP condensate incubated with TAMRA‐labelled DNA. The color scale (right) represents fluorescence lifetime in nanoseconds (ns). Scale bars, 10 µM. (E) Schematic illustrating the measurement of fluorescence lifetime inside versus outside the condensates. (F) Quantification of GBF1‐GFP fluorescence lifetime when incubated with no DNA, *GBOXm*, or *GBOX*. Data are presented as mean ± standard deviation (SD, *n* = 6). ^***^ *p* < 0.001, ^**^ *p* < 0.01, ^*^ *p* < 0.05 (two‐tailed Student's *t*‐test). (G) A proposed model showing that condensation of GBF1 enriches its dimeric form, thereby facilitating the binding to the target G‐box DNA.

The bZIP proteins are known to recognize and bind the G‐box motif in DNA [34]. GBF1 was reported to bind a G‐box motif within the promoter of the *CathB3* gene [33], which encodes Cathepsin B‐like cysteine protease responsible for seed storage protein degradation and mobilization during seed germination [35, 36]. We synthesized a 30‐bp double‐stranded DNA fragment (*GBOX*) from the *CathB3* promoter encompassing the G‐box and labeled it with TAMRA fluorophore at the 5’ end (Figure 2A). An electrophoretic mobility shift assay (EMSA) confirmed the binding of GBF1 with *GBO* *X* (Figure S3A). As a control, *GBOXm* with the binding site mutated was not bound by GBF1 (Figure 2A; Figure S3A), indicating that GBF1 binding to target DNA is specific. When incubated with GBF1‐GFP condensates in vitro, both *GBO* *X* and *GBO* *X* *m* were partitioned into the condensates (Figure 2B), suggesting that the recruitment into the condensates does not reflect specific binding. To examine the impact of GBF1 condensation on its DNA binding, we employed fluorescence lifetime microscopy and Förster Resonance Energy Transfer (FLIM‐FRET) assay, in which shorter donor lifetime indicates stronger binding (Figure 2C). Indeed, a much shorter GFP lifetime was observed when GBF1‐GFP was incubated with *GBO* *X* compared to *GBO* *Xm* or no DNA control (Figure 2D). We then quantified GFP lifetime inside and outside GBF1 condensates to compare the binding efficiency (Figure 2E). The results showed significantly shorter lifetimes inside than outside (Figure 2F), suggesting that GBF1 binds more efficiently to target DNA within the condensates. In line with this observation, EMSA revealed that the non‐condensing variant GBF1^∆PrLD^ bound less to *GBO* *X* compared to wild‐type GBF1 (Figure S3B). As GBF1 binds target DNA as a dimer, and dimerization is required for GBF1 condensation, we propose that GBF1 condensation enhances DNA binding by enriching dimers upon equilibrium (Figure 2G).

### 2.4 GBF1 Condensation is Indispensable for its Transcriptional Activity

**FIGURE 3:**
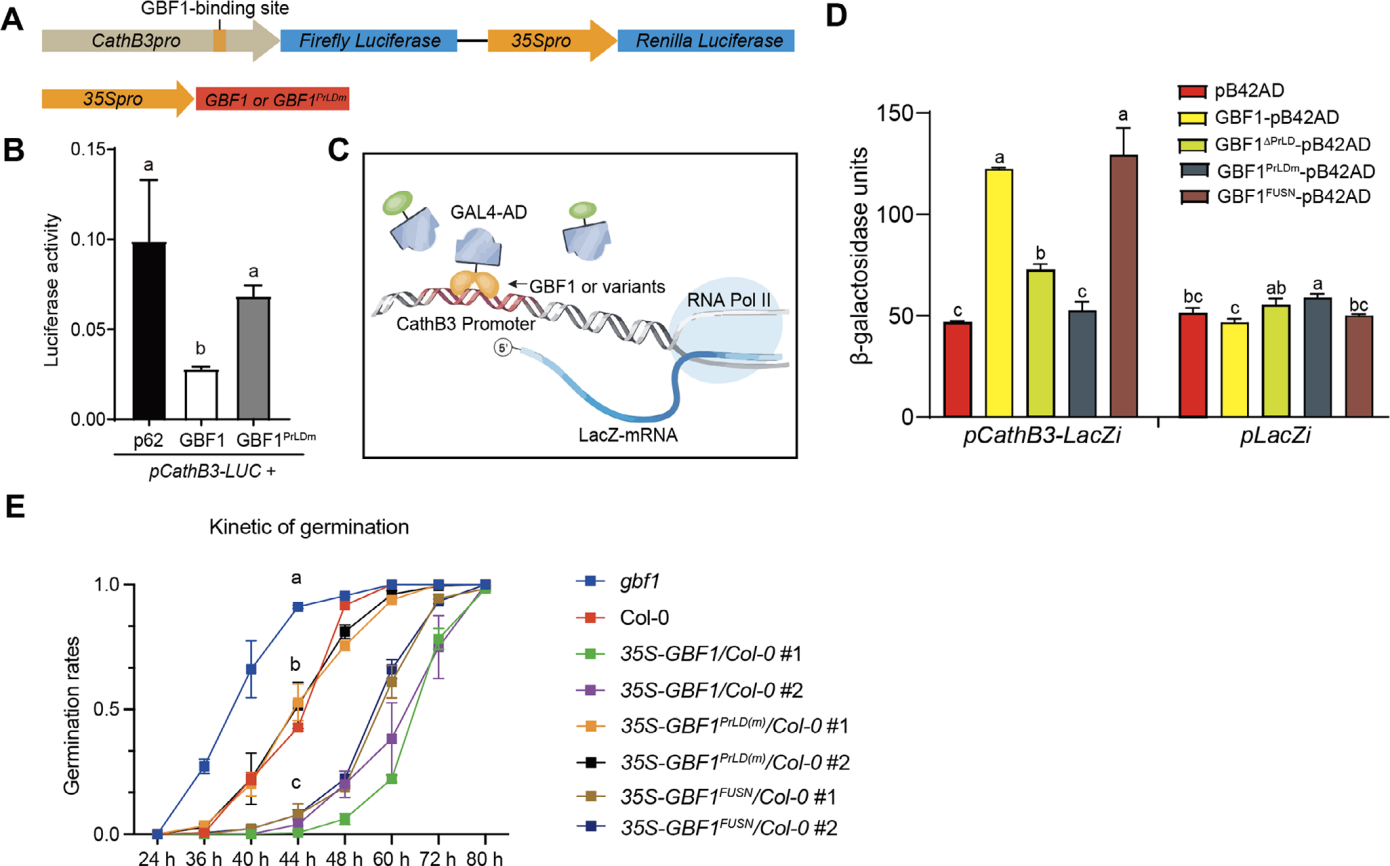
Phase separation of GBF1 enhances its activity. (A) Schematic of the dual‐luciferase reporters for transcriptional activity assay in Arabidopsis protoplasts. (B) Luciferase activity driven by the *CathB3* promoter upon co‐expression with the indicated GBF1 variants or empty vector (p62). (C) Schematic of the β‐galactosidase reporter assay to test DNA binding in yeast cells. (D) The β‐galactosidase activity upon co‐expression of the *pCathB3::LacZ* reporter with the indicated GBF1‐AD (Activation Domain) fusion proteins or pB42‐AD control in yeast cells. Data (in B, D) are mean ± SD (*n* = 3). Different letters indicate statistically significant differences (*p* < 0.05, one‐way ANOVA with Tukey's post‐hoc test). (E) Germination kinetics of the indicated genotypes. Data are presented as mean ± SEM (standard error of the mean，*n* = 3 independent replicates; ≥ 80 seeds per replicate). Groups with different letters indicate significant differences (*p* < 0.05, one‐way ANOVA with Tukey's post‐hoc test).

We next investigated how condensation affects the activity of GBF1. We observed that in the yeast two‐hybrid system, when GBF1 was fused to the GAL4 DNA‐binding domain, it exhibited self‐activation (Figure S3C). However, self‐activation was abolished when the PrLD was either deleted or mutated, and was retained when the PrLD was replaced with FUSN (Figure S3C), supporting the notion that the phase‐separation capacity is required for the transcriptional activity of GBF1. To confirm this, we performed a dual‐luciferase reporter assay in Arabidopsis protoplasts. The *CathB3* promoter was used to drive the firefly luciferase (LUC) reporter gene, with a 35S‐driven Renilla luciferase (REN) serving as an internal control for normalization (Figure 3A). Co‐expression with wild‐type GBF1 dramatically reduced luciferase activity compared to that of p62 (Figure 3B). In contrast, co‐expression of GBF1^PrLDm^ had a negligible effect (Figure 3B). To more directly test whether the phase separation affects the DNA‐binding of GBF1 to target DNA in cells, we inserted the *CathB3* promoter into a construct to drive the expression of the *LacZ* gene, which encodes β‐galactosidase. GBF1 or its variants were fused with the B42 transcriptional activation domain (AD). Therefore, the activity of beta‐galactosidase reports the DNA‐binding capacity (Figure 3C). The results showed that compared to p62‐AD, which does not bind the *CathB3* promoter, the condensing GBF1 activated *LacZ* gene expression, whereas the condensation‐deficient variants showed much less or no activation (Figure 3D). GBF1 was reported to inhibit seed germination; consequently, overexpression of GBF1 led to delayed germination [33]. We found that significantly delayed germination was caused by overexpressing condensing versions of GBF1 but not by overexpressing GBF1^PrLDm^ (Figure 3E; Figure S3D). Collectively, these results demonstrate that the in vivo activity of GBF1 is dependent on its ability to form biomolecular condensates.

### 2.5 GIP1 Functions Together With GBF1 During Seed Germination

**FIGURE 4:**
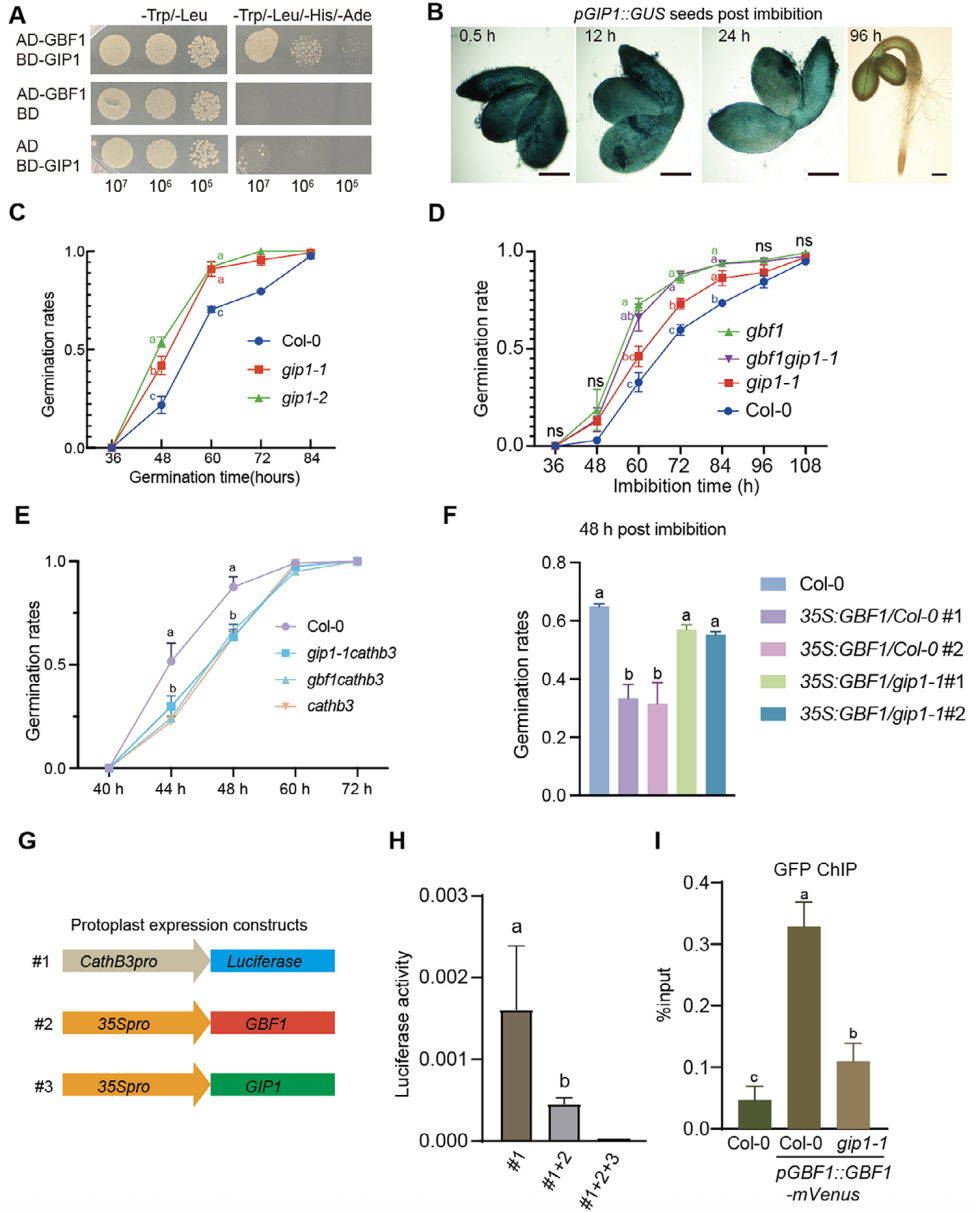
GIP1 interacts with GBF1 and enhances GBF1 function during seed germination. (A) Yeast two‐hybrid assay showing the interaction between GBF1 and GIP1. (B) Histochemical GUS staining of imbibed seeds from *pGIP1::GUS* transgenic plants. Scale bars, 200 µM. (C–E) Germination kinetics of the indicated genotypes. Data are mean ± SEM (*n* = 3 independent replicates; ≥ 80 seeds per replicate). (F) Germination rates of the indicated genotypes at 48 h after imbibition. Data are presented as mean ± SEM (*n* = 3 independent replicates; ≥ 80 seeds per replicate). Groups with different letters indicate significant differences (*p* < 0.05, one‐way ANOVA with Tukey's post‐hoc test). (G) Schematic of the dual‐luciferase reporter constructs. (H) Luciferase activity driven by the *CathB3* promoter upon co‐expression with the indicated constructs in Arabidopsis protoplasts. (I) In vivo chromatin immunoprecipitation (ChIP)‐qPCR analysis of GBF1 binding to the *CathB3* promoter in wild‐type (Col‐0) and *gip1‐1* mutant seeds. Data are presented as a percentage of input. Data (in H and I) are mean ± SD (*n* = 3). Groups with different letters indicate significant differences (*p* < 0.05, one‐way ANOVA with Tukey's post‐hoc test).

GIP1 was previously identified using maize GBF1 as bait in a yeast two‐hybrid screen of an Arabidopsis cDNA library [37]. It was found to enhance the DNA‐binding activity of Arabidopsis GBF3 or maize GBF1 [37], but the underlying mechanism is not fully understood. Our yeast two‐hybrid experiment confirmed the interaction of GBF1 with GIP1 (Figure 4A). Our histochemical staining of GUS driven by the GIP1 promoter showed that, similar to GBF1, GIP1 was highly expressed in the germinating seed embryos (Figure 4B; Figure S4B), prompting us to test its role in seed germination. We obtained a T‐DNA insertion mutant (*gip1‐1*) with reduced *GIP1* expression (Figure S4A,B) and a second CRISPR‐Cas9 allele (*gip1‐2*) carrying a genomic deletion that introduces a premature stop codon (Figure S4A,C). Both *gip1* mutants germinated earlier than Col‐0 (Figure 4C), indicating that GIP1 represses germination. We further examined *gbf1*, *gip1‐1*, and *gbf1 gip1‐1* double mutants. The double mutant phenocopied *gbf1*, not *gip1*, placing *GBF1* genetically downstream of *GIP1* (Figure 4D). We next found that both the *gip1‐1 cathb3* and *gbf1 cathb3* double mutants phenocopied the *cathb3* single mutant, exhibiting delayed germination (Figure 4E). These results indicate that *CathB3* acts genetically downstream of *GIP1* and *GBF1*. Importantly, overexpression of GBF1 repressed seed germination and *CathB3* expression in Col‐0 but not in the *gip1‐1* mutant (Figure 4F; Figure S4G), indicating that the germination‐repressing function of GBF1 and its transcriptional repression of *CathB3* are both dependent on GIP1.

Moreover, transient expression assays in protoplasts demonstrated that GIP1 enhances GBF1‐mediated repression of a *CathB3* promoter‐driven reporter (Figure 4G,H). In vivo ChIP‐qPCR showed that GBF1 associated with the promoter of *CathB3*, and this association was reduced in the *gip1‐1* mutant (Figure 4I). These results demonstrate that GIP1 functions during seed germination by positively regulating the activity of GBF1.

### 2.6 GIP1 Regulates the Size and Material Properties of GBF1 Condensates

**FIGURE 5:**
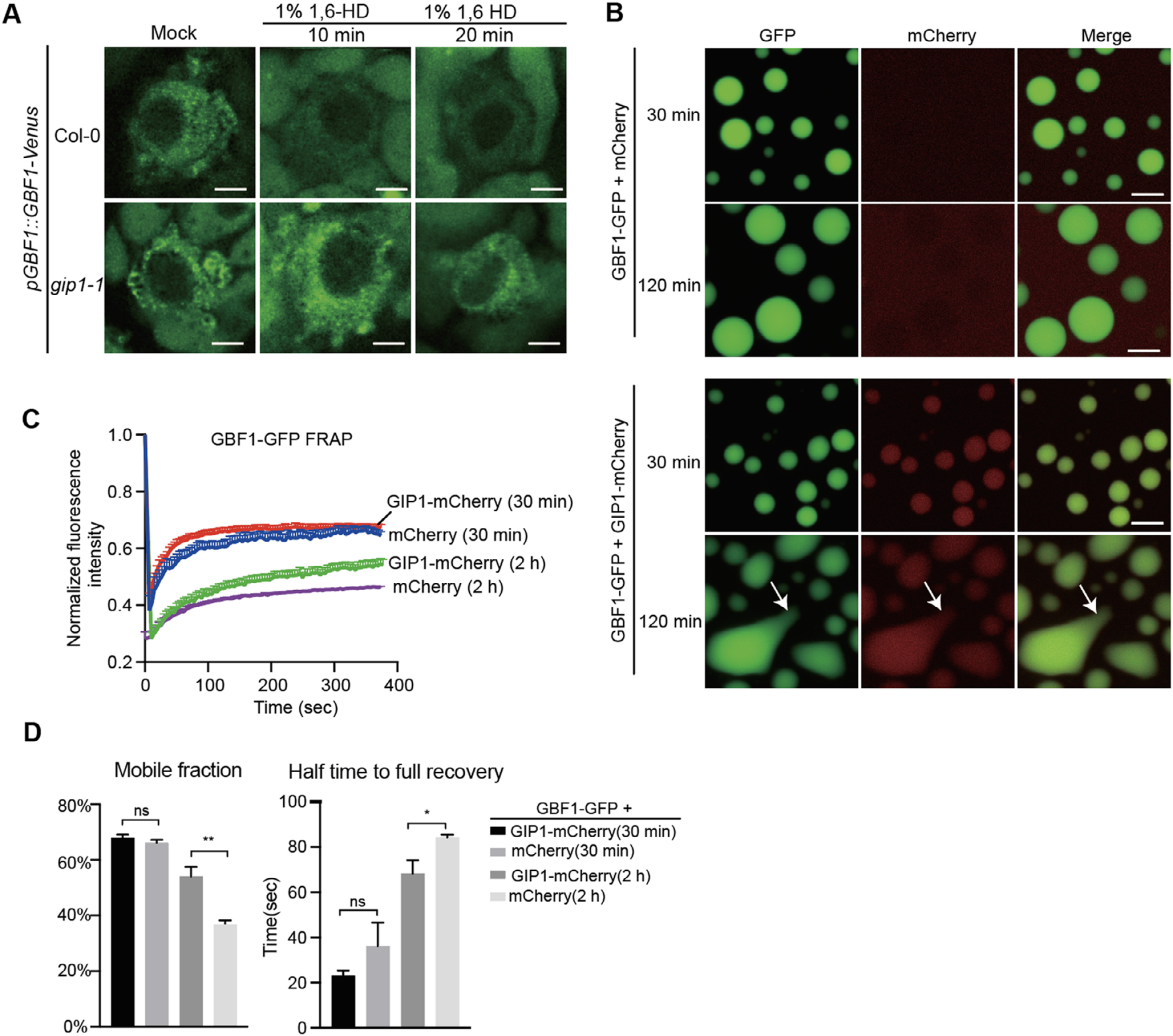
GIP1 regulates the size and material properties of GBF1 condensates. (A) Sensitivity of GBF1‐mVenus condensates in seed embryos to 1% 1,6‐hexanediol (1,6‐HD) treatment for the indicated time in wild‐type (Col‐0) and *gip1‐1* mutant backgrounds. Scale bars, 2 µM. (B) Time‐lapse imaging of GBF1‐GFP condensates in vitro in the presence or absence of GIP1‐mCherry. Scale bars, 5 µM. (C) FRAP analysis of GBF1‐GFP condensates in vitro with or without GIP1. (D) Mobile fraction (left) and half‐time of recovery (t_1_/_2_, right) of GBF1 condensates in the absence or presence of GIP1. Data represent mean ± SD (*n* = 3). Statistical significance was determined by unpaired two‑tailed Student's *t*‑test (^***^ *p* < 0.001, ^**^ *p* < 0.01, ^*^ *p* < 0.05; ns, not significant).

An earlier study proposed GIP1 as a chaperone, but the mechanism of chaperoning is unclear [37]. Given the functional dependence of GBF1 on condensation and its co‐enrichment with GIP1 by b‐isox (Figure S1A), we hypothesized a potential functional interplay between GIP1 and GBF1 condensates. In vitro, GIP1 alone did not form condensates (Figure S5A), but was localized into GBF1 condensates when mixed together (Figure S5B). Notably, the addition of GIP1 reduced both the size and number of GBF1 condensates (Figure S5B–D). Consistently, the presence of GIP1 reduced the turbidity of the GBF1 protein solution to transparency (Figure S5E,F). These observations suggest that GIP1 might behave like a surfactant, adsorbing onto biological condensates to modify the interface and reduce interfacial tension, thus regulating the size and structure of the condensates [38, 39]. Indeed, time‐lapse imaging revealed that GIP1 was initially attached to the surface of GBF1 condensates and subsequently diffused into them (Figure S5G). To assess the physiological relevance of GIP1's effect on condensate size, we compared GBF1 condensates in wild‐type and *gip1‐1* mutant seed embryos. However, due to the light diffraction limit, we were unable to conclude any difference in the size of GBF1 condensates (Figure 5A).

In the phase‐separated system, coarsening is a fundamental process that increases the average size of the distinct phases [40, 41]. We observed GBF1‐GFP condensates in vitro for over 2 h and found that the size of the GBF1 condensates increased regardless of whether GIP1 was present or not (Figure 5B). However, we noticed that in the presence of GIP1, GBF1 condensates wetted the slide more than those without GIP1 (Figure 5B), suggesting higher liquidity. We performed photobleaching and found that GBF1‐GFP fluorescence recovered better when GIP1 was present (Figure 5C). Quantitative analysis further revealed that GIP1 significantly increased the mobile fraction and decreased the half‑time of recovery (t_1_/_2_) of GBF1 condensates (Figure 5D), confirming that GIP1 enhances molecular dynamics within the condensates. To assess the material properties of GBF1 condensates in vivo, we treated seed embryos with a low concentration of 1,6‐hexanediol, which dissolves liquid‐like condensates [42]. The results showed that GBF1 condensates in the *gip1‐1* mutant background were more resistant to 1,6‐hexanediol (Figure 5A). These results indicate that GIP1 chaperones the size and liquidity of GBF1 condensates, preventing them from growing too large and becoming solid. Next, we found that solidification directly impairs DNA binding. We used FLIM‐FRET to measure the DNA binding within the condensed phases of both liquid and aged (solid) GBF1 condensates in vitro. Although target DNA was similarly recruited, FLIM‑FRET showed that liquid condensates exhibited higher DNA‐binding affinity than the aged (solid) GBF1 condensates (Figure S6). The loss of DNA binding in solid GBF1 condensates indicates that liquidity is essential for productive target binding.

### 2.7 The Function of GIP1 Is Redox‐Sensitive

**FIGURE 6:**
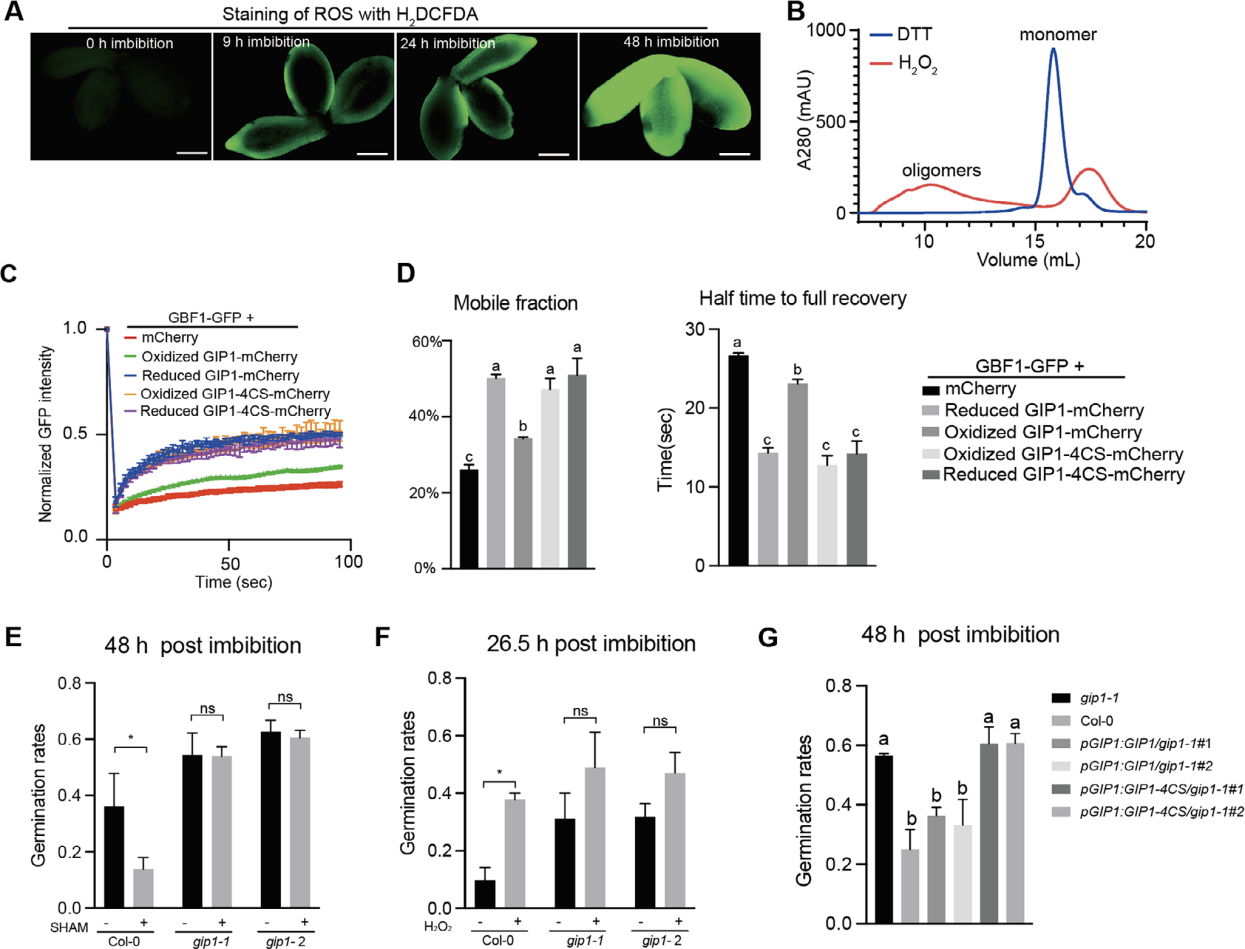
The chaperone activity of GIP1 is sensitive to redox conditions. (A) ROS detection using H_2_DCFDA fluorescence in wild‐type (Col‐0) seeds during imbibition. Scale bars, 200 µM. (B) Size exclusion chromatography profiles of GIP1 under reducing (5 mm DTT) and oxidizing (3 mm H_2_O_2_) conditions. (C) FRAP of GBF1 condensates treated with reduced GIP1, oxidized GIP1, or GIP1‐4CS. (D) Comparison of mobile fraction (left) and half‐time of recovery (t_1_/_2_, right) of GBF1 condensates treated with indicated forms of GIP1. Data are shown as mean ± SD (*n* = 3). Groups with different letters indicate significant differences (*p* < 0.05, one‐way ANOVA with Tukey's post‐hoc test). (E) Germination rates of Col‐0, *gip1‐1*, and *gip1‐2* under normal conditions (1/2 MS) and after treatment with 400 µm SHAM for 48 h. (F) Germination rates of the indicated genotypes at 26.5 h after pretreatment with 25 mm H_2_O_2_. (G) Germination rates of the indicated genotypes at 48 hour under normal conditions. Data (in E–G) are mean ± SEM (*n* = 3 independent replicates; ≥ 80 seeds per replicate). Groups with different letters indicate significant differences (*p* < 0.05, one‐way ANOVA with Tukey's post‐hoc test).

ROS are pivotal for initiating seed germination [5, 43]. We monitored spatial and temporal ROS dynamics during Arabidopsis seed imbibition using the fluorescent probe 2’,7’‐dichlorodihydrofluorescein diacetate (H_2_DCFDA). Indeed, a rapid accumulation of ROS signal was observed upon imbibition (Figure 6A), which coincided with the expression pattern of GIP1 as revealed by GUS staining (Figure 4B). Analysis of the GIP1 protein sequence showed that it contains four cysteine residues (Figure S7A), which are known targets of ROS and form either inter‐ or intramolecular disulfide bonds [44]. We therefore examined the state of GIP1 under oxidized and reduced conditions in vitro. Using non‐reduced sodium dodecyl sulfate polyacrylamide gel electrophoresis (non‐reduced SDS‐PAGE), in which the reducing agent β‐mercaptoethanol is omitted to preserve native disulfide bonds, we found that GIP1 migrated primarily as oligomers upon H_2_O_2_ treatment, which was reduced to monomers by Dithiothreitol (DTT) (Figure S7B). Consistent with this, size‐exclusion chromatography revealed a pronounced shift toward higher‐molecular‐weight oligomers under oxidizing conditions (Figure 6B), demonstrating that GIP1 oligomerizes via intermolecular disulfide bonds. We next generated the GIP1‐4CS mutant by substituting four cysteine residues with serine to investigate whether the oxidized or the reduced form of GIP1 is functional in regulating GBF1. Yeast two‑hybrid assays indicated that the cysteine mutations did not disrupt the interaction with GBF1 (Figure S7C). Oxidation abolished GIP1's ability to regulate GBF1 condensate size, number, and dynamics, but not that of GIP1‐4CS (Figure 6C,D; Figure S7D,E), indicating that the cysteine residues are crucial for redox sensing by GIP1.

The above observations suggest a model in which GIP1 senses ROS in seeds and regulates germination by fine‐tuning GBF1 condensation. To test this, we manipulated ROS levels by exogenously applying salicylhydroxamic acid (SHAM), a peroxidase inhibitor that scavenges endogenous H_2_O_2_, thereby reducing cellular ROS levels [45]. SHAM treatment significantly suppressed germination in wild‐type Col‐0 seeds (Figure 6E). In contrast, the *gip1* mutants showed markedly reduced sensitivity to SHAM inhibition (Figure 6E). Conversely, exogenous application of H_2_O_2_ accelerated germination of wild‐type Col‐0 seeds but not those of the *gip1* mutants (Figure 6F). To further investigate if the oxidation of cysteine residues of GIP1 confers ROS sensing, we expressed wild‐type GIP1 or redox‐insensitive GIP1‐4CS under the native promoter in the *gip1‐1* mutant background. The results showed that wild‐type GIP1 fully restored the early germination phenotype of the *gip1‐1* mutant, whereas GIP1‐4CS did not (Figure 6G). Similarly, *pGIP1::GIP1‐4CS/gip1‐1* behaved similarly to the *gip1‐1* mutant upon SHAM or H_2_O_2_ treatment (Figure S7F).

## 3 Discussion

**FIGURE 7:**
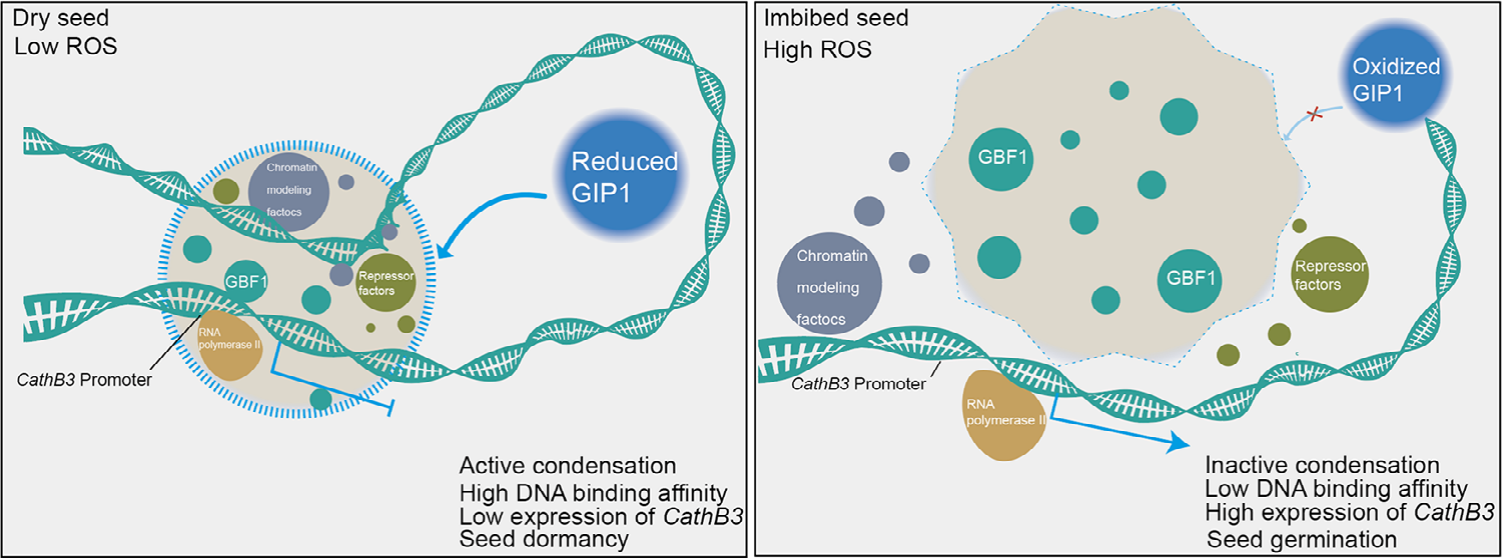
Working model: A redox‐sensitive chaperone‐condensate axis gates seed germination. In dormant seeds (low ROS), reduced GIP1 chaperones GBF1 condensates, maintaining their liquid‐like state, enhancing DNA binding, and promoting repression of the germination gene *CathB3*. Upon imbibition (high ROS), ROS oxidize GIP1, impairing its chaperone activity. This leads to the maturation of GBF1 condensates into less dynamic, solid‐like states, reducing DNA binding and derepressing *CathB3*, thereby permitting germination.

Seed germination is governed by a balance between environmental cues and intrinsic developmental programs, yet the mechanisms that translate redox signals into transcriptional regulation have remained elusive. Our study identifies a redox‐sensitive chaperone‐condensate module as a regulator in this process. We show that the transcription factor GBF1 forms functional condensates that repress the germination‐promoting gene *CathB3*, and that its repressive activity depends on the liquidity of these assemblies. GIP1 functions as a molecular chaperone that tunes the size and fluidity of GBF1 condensates, thereby enhancing DNA binding and transcriptional repression. Crucially, ROS oxidize GIP1 and abolish its chaperone activity, leading to aberrant condensate maturation and derepression of *CathB3*, which promotes germination (Figure 7).

We demonstrate that GBF1 undergoes LLPS both in vitro and in vivo, forming dynamic nuclear condensates in seed embryos. These condensates depend on multivalent interactions mediated by the PrLD and the dimerization‐capable bZIP DNA‐binding domain. Importantly, condensation enhances GBF1's binding to the G‐box motif in the *CathB3* promoter. We proposed that this resulted from the enrichment of dimerization by condensation, which is a prerequisite for target DNA binding. Further experiments are still needed to test this hypothesis. The condensation of transcription factors was found to enrich the transcription apparatus [11], enhance target search [46], or sequester itself away from the transcription site [47]. GBF1 condensation may have multiple effects on transcription. Other families of transcription factors, such as TCPs, also require dimerization to bind DNA [48]; it is worth testing whether they also function via biomolecular condensation.

We found that liquid condensates exhibited higher DNA‐binding affinity than the aged (solid) GBF1 condensates (Figure S6). We therefore propose that conformational restriction and loss of dynamic exchange may explain this difference. GBF1 requires dimerization to efficiently bind the G‐box motif. Liquid condensates permit frequent molecular exchange and conformational rearrangements, facilitating dimer formation and target association. Solid condensates lock proteins into a rigid network, thereby restricting dimer dynamics and conformational flexibility and reducing specific binding affinity.

These findings advance our understanding of how condensate material properties integrate with environmental signals to regulate developmental transitions. While previous studies established that transcription factors can form condensates to activate or repress gene expression, our work demonstrates that the outcome is not determined by condensation per se, but by whether these condensates remain dynamic. The chaperones, including heat shock proteins, are emerging as regulators of the material properties of biomolecular condensates in yeast and animal systems [49, 50, 51]. The discovery that a chaperone safeguards condensate liquidity in plants highlights a widespread regulatory layer in which chaperones extend their influence beyond proteostasis into controlling the physical properties of condensates. Yet the mechanism underlying the chaperone activity of GIP1 needs further investigation.

More broadly, the GIP1‐GBF1 module illustrates how plants harness biophysical principles to convert redox cues into developmental outcomes. Given the conservation of both bZIP transcription factors and chaperone‐like proteins across eukaryotes, analogous mechanisms may regulate phase separation in other systems. Future work should explore whether other redox‐sensitive chaperones act on transcriptional condensates and whether such regulation extends to additional developmental processes or stress responses.

In summary, our study reveals that ROS‐dependent chaperoning of transcriptional condensates constitutes a critical regulatory node controlling seed germination. By linking environmental redox signals to the material properties of condensates, this mechanism establishes a paradigm for how plants couple phase separation dynamics to developmental decisions.

## 4 Materials and Methods

### 4.1 Plant Materials and Growth Conditions

All the *Arabidopsis thaliana* mutants and transgenic plants used in this study were in the Col‐0 background. The T‐DNA insertion mutants *gip1‐1* (SALK_123671, insertion in the 5′ UTR) and *cathb3* (WiscDsLox336C03, insertion in the 5′ UTR) were obtained from the AraShare, and *gbf1* (SALK_144534, insertion in the exon 5) was obtained from the Arabidopsis Biological Resource Center (ABRC). The *gip1‐1 gbf1, gip1‐1 cathb3, and gbf1 cathb3* double mutants were generated by genetic crossing of the respective single mutants. To generate the *gip1‐2* CRISPR allele, two sgRNAs targeting exon 1 and exon 3 of *GIP1* were designed and cloned into a CRISPR‐Cas9 vector. Transgenic plants were selected, and the 818 bp deletion was confirmed by PCR and Sanger sequencing. Two sgRNAs targeting sequences are listed in Table S1.

Seeds were surface‐sterilized with 75% ethanol for 5 min, followed by three washes with sterile water, and then treated with 10% sodium hypochlorite containing 0.1% Tween‐20 for 3–5 min. After five additional washes with sterile water, seeds were sown on half‐strength Murashige and Skoog (1/2 MS) medium containing 0.8% (w/v) agar and 1% (w/v) sucrose. Plates were stratified at 4°C in the dark for 3 days and subsequently transferred to a growth chamber. Plants were grown under long‐day conditions (16 h light/8 h dark) at 20°C–22°C with a light intensity of 100–150 µMol m^−2^ s^−1^. Plate media were transferred to a growth chamber after about 7 days. *Nicotiana benthamiana* plants were grown in soil under similar conditions for 3–4 weeks before using in transient expression assays.

### 4.2 Germination Phenotype Analysis

To ensure uniformity in seed physiological status, all seeds used in germination assays were from plants grown and harvested simultaneously, followed by an after‐ripening period of more than three months under dry ambient conditions to break dormancy. Mature, after‐ripened seeds from different genotypes were sown on 1/2 MS agar plates. Plates were transferred to a growth chamber under standard conditions. Germination, defined as radicle emergence through the seed coat, was scored every 4h or 12 h. For each experiment, a minimum of three biological replicates were performed, with each replicate consisting of 80–100 seeds per genotype and condition.

For ROS manipulation assays, seeds were germinated on plates supplemented with 400 µm SHAM, a peroxidase inhibitor. Alternatively, seeds of different genotypes were soaked overnight in liquid 1/2 MS medium with 25 mm H_2_O_2_ and kept in the dark to assess exogenous H_2_O_2_. The following morning, the treated seeds were blotted dry and sown on standard 1/2 MS agar plates for germination scoring. For each experiment, a minimum of three biological replicates were performed, with each replicate consisting of 80–100 seeds per genotype and condition.

### 4.3 Genotyping

Genomic DNA was extracted from leaf tissue. T‐DNA insertion lines were genotyped by PCR using gene‐specific primers in combination with T‐DNA left border primer LBb1.3. Genotyping of *gip1‐2* was performed using primers, yielding a wild‐type band of about 3000 bp and a deletion band of about 2000 bp. Primer sequences used for genotyping are listed in Table S1. PCR products were analyzed by agarose gel electrophoresis.

### 4.4 Molecular Cloning

The CDS of *GIP1*, *bZIP16*, *bZIP68*, and *GBF1* (and their variants) were amplified from Arabidopsis cDNA using Phusion High‐Fidelity DNA Polymerase (Thermo Fisher Scientific) or Platinum SuperFi II DNA Polymerase (Thermo Fisher Scientific). Amplified fragments were cloned into appropriate destination vectors (e.g., pCAMBIA1300 for plant transformation, pET‐based vectors for bacterial expression, pDUAL for yeast expression, pB42AD and pLacZi for β‐Galactosidase assay, pADT7 and pBKT7 for yeast one‐ or two‐hybrid assay) using either restriction enzyme‐based cloning or the ClonExpress Ultra One Step Cloning Kit (Vazyme, C115). Site‐directed mutagenesis (e.g., *GIP1‐4CS*, *GBF1* ^*PrLDm*^) was performed using a standard two‐step PCR method or Chemical synthesis. All constructs were verified by Sanger sequencing. Primer sequences are available in Table S1.

### 4.5 Plasmid Construct

The *pGBF1::GBF1‐mVenus* construct was generated by amplifying a 1.8‐kb promoter region upstream of the *GBF1* start codon from wild‐type Col‐0 genomic DNA and cloning it into the pCAMBIA1300‐mVenus vector, resulting in an intermediate *pGBF1::mVenus* vector. The CDS of *GBF1* was then inserted between the promoter and the *mVenus* tag. Using the same vector backbone and a similar strategy, we also generated the *pGIP1::GIP1‐mVenus* construct, in which the *GIP1* CDS was placed under the control of its 1.6‐kbp promoter. Additionally, a redox‐insensitive version, *pGIP1::GIP1‐4CS‐mVenus*, was created by introducing point mutations (cysteine‐to‐serine substitutions at four conserved residues) via site‐directed mutagenesis and overlap extension PCR.

To generate the constructs used for in vitro protein expression, the following procedures were employed in pET‐based vectors. The CDS for *GBF1^ΔPrLD^*, *GBF1^ΔIDR^*, and *GBF1^ΔbZIP^* were generated using a standard two‐step PCR method to remove the respective domains (PrLD, IDR, or bZIP domain) and verified by DNA sequencing. To generate the *GBF1^FUSN^* variant, the *FUSN* domain was cloned and fused to the *GBF1^ΔPrLD^* CDS via overlap extension PCR. Similarly, the *GBF1^PrLDm^* variant was produced by synthesizing a mutated *PrLD* sequence (phenylalanine and tyrosine‐to‐serine substitutions at 11 conserved residues) and fusing it to the *GBF1^ΔPrLD^* CDS using overlap extension PCR. The CDS of *GBF1* and its variants (*GBF1^ΔIDR^*, *GBF1^ΔbZIP^*, *GBF1^ΔPrLD^*, *GBF1^PrLDm^*, and *GBF1^FUSN^*), *GIP1* and its variants (*GIP‐4CS*), were amplified and inserted into the pET11‐6×His‐GFP, pET11‐6×His‐mCherry expression vector (digested with BamHI). To generate the in vitro purified GBF1 without GFP tag, pET11‐6×His‐GFP was digested by BamHI and XhoI to remove GFP and insert *GBF1* CDS. Where necessary, a maltose‐binding protein (MBP) solubility tag was placed at the N‐terminus of the construct, followed by a tobacco etch virus protease (TEV) cleavage site.

For plant transformation, *GBF1*, *GBF1^ΔPrLD^*, *GBF1^PrLDm^*, and *GBF1^FUSN^* fragments were subsequently inserted between the *35S* promoter and the *mVenus* coding sequence in the pCAMBIA1300‐35S‐mVenus vector via homologous recombination*‐*. Using the same vector backbone and a similar strategy, we also generated the *35S::GIP1‐mVenus* construct, in which the *GIP1* CDS was placed under the control of the 35S promoter.

To generate the constructs used for heterologous expression in yeast cells, the CDS of GBF1 and its variants (*GBF1^ΔIDR^*, *GBF1^ΔbZIP^*, *GBF1^ΔPrLD^*, *GBF1^PrLDm^*, and *GBF1^FUSN^*) were amplified and inserted into the pDUAL‐Pnmt1‐yeGFP vector (digested with NheI and BamHI).

To generate fluorescently tagged constructs for protein localization in *N. benthamiana* epidermal cells, the CDS of *bZIP16* and *bZIP68* were individually cloned into plant expression vectors under the control of the *35S* promoter. Specifically, the *bZIP16* CDS was inserted into the pCAMBIA1300‐35S‐mCerulean vector using the SalI restriction site, creating *35S::bZIP16‐mCerulean* construct. Similarly, the *bZIP68* CDS was cloned into the pCAMBIA1300‐35S‐mScarlet‐I vector using the BamHI site, resulting in the *35S::bZIP68‐mScarlet‐I* construct.

To analyze the transcriptional repression activity of the GBF1, constructs for expression in protoplasts were produced. A 1.1‐kbp genomic fragment spanning from −1162 bp to the translation start site (ATG) was amplified and inserted into the pGreenII 0800‐LUC vector via the BamHI and SpeI restriction sites, resulting in the reporter construct *pCathB3::LUC*. This plasmid contains a LUC gene driven by the *CathB3* promoter and an internal control REN gene under a constitutive promoter. For effector constructs, the CDS of *GBF1*, *GBF1* ^PrLDm^, and *GIP1* were amplified and inserted downstream of the 35S promoter in the pGreenII 62‐SK vector using the SpeI and BamHI restriction sites. This resulted in the *35S::GBF1, 35S::GBF1^PrLDm^*, and *35S::GIP1* overexpression constructs, respectively. These constructs were subsequently used in transient transfection assays of Arabidopsis mesophyll protoplasts.

For the β‐galactosidase activity assay, the CDS of *GBF1* and its variants (*GBF1* ^*PrLDm*^, *GBF1* ^*FUSN*^, and *GBF1* ^*ΔPrLD*^) were amplified and inserted into the pB42AD vector using the EcoRI and XhoI restriction sites, resulting in in‐frame fusions downstream of the B42 transcriptional activation domain. For the reporter construct, a 1.1‐kbp DNA fragment of the *CathB3* promoter was cloned into the pLacZi vector via the KpnI and XhoI sites, placing it upstream of the *lacZ* reporter gene.

To assess the transcriptional activation capacity of GBF1 and its phase separation‐related variants, a yeast one‐hybrid assay was employed. The coding sequences of full‐length and mutant versions of *GBF1* (*GBF1* ^*PrLDm*^, *GBF1* ^*FUSN*^, and *GBF1* ^*ΔPrLD*^) were amplified and inserted downstream of the GAL4 DNA‐binding domain (BD) coding sequence in the pGBKT7 vector using the EcoRI and BamHI restriction sites. This resulted in the generation of in‐frame GAL4 BD‐GBF1 fusion constructs (*pBKT7‐GBF1*, *pBKT7‐GBF1^PrLDm^*, *pBKT7‐GBF1^FUSN^, pBKT7‐GBF1^ΔPrLD^*).

To explore protein‐protein interactions, the CDS of *GIP1* was amplified and inserted into the pGADT7 vector using the EcoRI and BamHI restriction sites, creating a fusion with the GAL4 activation domain. Similarly, the CDS of *GBF1* was cloned into the pGBKT7 vector using the same restriction sites, generating a fusion with the GAL4 BD.

### 4.6 Plant Transformation

Binary vectors were introduced into the *Agrobacterium tumefaciens* strain GV3101. Arabidopsis plants (Col‐0, *gbf1*, *gip1‐1*) were transformed using the floral dip method. Transgenic T1 plants were selected on 1/2 MS plates containing 30 mg/L hygromycin (AMRESCO, K547). Homozygous T3 or T4 lines were used for experiments.

### 4.7 b‐isox Precipitation and Mass Spectrometry Analysis

The b‐isox precipitation and mass spectrometry data shown in Figure S1A were derived from our previously published dataset [19]. The detailed experimental procedures can be found in that reference.

### 4.8 RNA Extraction and Quantitative RT‐PCR (qRT‐PCR)

Total RNA was extracted from seeds or seedlings using TRIzol reagent (Invitrogen, 15596018) or the TIANGEN Plant RNA Kit (DP452), following the manufacturer's protocols. Contaminating genomic DNA was removed using DNase I (Promega, M6101). First‐strand cDNA was synthesized from 1 to 3 µg of total RNA using M‐MLV Reverse Transcriptase (Invitrogen, 28025013) with oligo(dT). qRT‐PCR was performed on a QuantStudio 1 Real‐Time PCR System (Applied Biosystems) using M5 HiPer SYBR Premix EsTaq (Mei5 Biotechnology). The *UBC21* (At5g25760) gene was used as an internal control for normalization. Gene expression levels were calculated using the 2^(‐ΔCt) method. Primer sequences used for qRT‐PCR are listed in Table S1.

### 4.9 ROS Detection

Seed ROS levels were detected using the fluorescent probe 2',7'‐dichlorodihydrofluorescein diacetate (H_2_DCFDA). Seeds were peeled and incubated in 10 µm H_2_DCFDA (dissolved in PBS from a 10 mm DMSO stock) for 15–30 min in the dark. After washing with PBS, fluorescence was observed and imaged using a fluorescence microscope (excitation/emission ∼502/529 nM).

### 4.10 GUS Staining

Histochemical GUS staining was performed using a GUS staining kit (Huayueyang, GT0391) according to the manufacturer's instructions. Tissues were incubated in GUS staining solution at 37°C overnight in the dark. Chlorophyll was cleared using 70%–75% ethanol at 65°C before observation and imaging.

### 4.11 Protein Expression and Purification

Recombinant proteins were expressed in *Escherichia coli* Rosetta (DE3) cells. Cultures were grown at 37°C to an OD600 of 0.6–0.8, and protein expression was induced with 0.4–0.5 mm isopropyl‐β‐D‐1‐thiogalactopyranoside (IPTG) at 16°C overnight. Cells were harvested by centrifugation at 4000 rpm for 15–20 min at 4°C, resuspended in lysis buffer (40 mm Tris‐HCl (pH 7.4), 1000 mm NaCl, 10% glycerol), and lysed by sonication. The lysate was clarified by centrifugation. The supernatant was incubated with Ni‐NTA beads 6FF (Smart‐lifesciences, Ni NTA Beads 6FF: SA00501L) pre‐equilibrated with lysis buffer. To minimize nucleic acid contamination, the resin was first washed extensively with a high‐salt buffer (40 mm Tris‐HCl, pH 7.4, 1 m NaCl, 20–30 mm imidazole), followed by washing with standard wash buffer (40 mm Tris‐HCl (pH 7.4), 500 mm NaCl, 20–30 mm imidazole). His‐tagged proteins were subsequently eluted with elution buffer (40 mm Tris‐HCl (pH 7.4), 500 mm NaCl, 250 mm imidazole). Eluted proteins were further purified by gel filtration chromatography (Superdex‐200; GE Healthcare) in storage buffer (40 mm Tris‐HCl(pH 7.4), 500 mm NaCl, 1 mm DTT). Protein concentrations were determined by spectrophotometry (NanoDrop One, Thermo Fisher Scientific). Where applicable, solubility tags (e.g., MBP) were removed by incubation with TEV protease for 1–2 h at 4°C.

### 4.12 In Vitro Phase Separation Assays

Purified proteins were diluted to the indicated concentrations in indicated salt buffer (typically 40 mm Tris‐HCl (pH 7.4), 150 mm NaCl, 1 mm DTT). For co‐condensation assays, proteins were mixed at specified ratios before dilution. Samples were incubated in 384‐well low‐binding microscopy plates (Greiner bio‐one, 781090) for 20–30 min at room temperature before imaging. Droplet formation was observed using a Nikon A1 HD25 or Zeiss LSM880 confocal microscope with a 63× or 100× oil immersion objective. Images were captured with excitation/emission settings appropriate for the fluorescent tags (GFP: 488 nM; mCherry: 561 nM; BFP: 405 nM; TAMRA: 561 nM).

### 4.13 FRAP

FRAP experiments were performed on confocal microscopes (Nikon A1 HD25). A defined region within a condensate was bleached using high‐intensity laser light. Recovery of fluorescence into the bleached area was monitored over time by capturing images at low laser intensity at regular intervals. Fluorescence intensity within the bleached region was normalized to the pre‐bleach intensity and to a reference unbleached region. Recovery curves were plotted and analyzed using ImageJ (Fiji) or the microscope's proprietary software.

### 4.14 Quantification of FRAP Data

FRAP data were quantified according to Carnell et al. [52]. The mobile fraction was determined from the plateau of the normalized recovery curve, and the half‐time of recovery (t_1_/_2_) was obtained by interpolation as the time at which fluorescence intensity reached 50% of the plateau value. All quantitative analyses were performed using at least three independent replicates, and data are presented as mean ± SD.

### 4.15 Yeast Transformation for Heterologous Expression

The plasmids were linearized with NotI, and the resulting fragments were gel‐purified and transformed into the fission yeast strain LD328 (genotype his3‐D1 leu1‐32). Briefly, yeast cells were cultured until the OD600 reached 0.4–0.8. For each reaction, 500 µL cultured cells were collected, washed three times with sterilized water, and resuspended in buffer I (240 µL of 50% PEG3350, 36 µL of 1.0 m LiAc, and 50 µL of 2.0 mg/mL carrier DNA). The linearized DNA (34 µL, up to 1 µg) was added to the resuspended cells, mixed vigorously, and incubated at 42°C for 40 min. The cells were collected and resuspended in 100 µL water and plated on EMM + HT (EMM medium supplemented with 45 mg/L histidine and 15 µm thiamine) plates. After incubation at 30°C for 2–3 days, individual colonies were selected on EMM + H (EMM medium supplemented with 45 mg/L histidine) plates. The cells were used for subsequent imaging analyses.

### 4.16 EMSA

TAMRA‐labeled double‐stranded DNA probes corresponding to the wild‐type or mutant *CathB3* promoter sequences were incubated with purified GBF1 protein in 1× EMSA buffer (25 mm HEPES (pH 8.0), 40 mm KCl, 5 mm MgCl_2_, 1 mm EDTA, 8% glycerol, 1 mm DTT) for 1 h at room temperature. Reactions were resolved on a pre‐run 5% native polyacrylamide gel in 0.5× TBE buffer at 100 V for 60–90 min. Gels were scanned using a Typhoon FLA9500 imager (Cytiva) with the TAMRA channel. The probe sequence is available in Table S1.

### 4.17 ChIP‐qPCR

ChIP assays were performed using 24‐h imbibed seeds (Col‐0, *pGBF1::GBF1‐mVenus/Col‐0*, *pGBF1::GBF1‐mVenus/gip1‐1*) grown on filter paper over 1/2 MS medium. Harvested samples were cross‐linked with 1% formaldehyde in PBS under vacuum for 15 min. The reaction was quenched with 0.125 m glycine. After washing, tissues were frozen in liquid nitrogen, ground to powder, and stored at −80°C. For chromatin preparation, powder was homogenized in Honda buffer (0.44 m sucrose, 1.25% Ficoll, 2.5% Dextran T40, 20 mm HEPES‐KOH (pH 7.4), 10 mm MgCl_2_, 0.5% Triton X‐100) supplemented with 10 mm DTT and 1× protease inhibitor cocktail, filtered through Miracloth (Millipore, 475855‐1RCN), and centrifuged at 4000 ×g for 5 min at 4°C. The pellet was lysed in nuclear lysis buffer (50 mm Tris‐HCl (pH 7.5), 10 mm EDTA, 0.5% SDS) with 2× protease inhibitor cocktail, then sonicated (15 min, 5 sec on/off, 60% amplitude). Cleared lysate was incubated overnight at 4°C with GFP‐Nanoab‐Magnetic Beads (LABLEAD, GNA‐50‐1000) pre‐washed in ChIP dilution buffer (50 mm Tris‐HCl (pH 7.5), 150 mm NaCl, 1 mm EDTA, 1% Triton X‐100, 0.1% SDS). Beads were washed sequentially with: Buffer A (150 mm NaCl, 20 mm Tris‐HCl (pH 7.5), 2 mm EDTA, 1% Triton X‐100); Buffer B (500 mm NaCl, 20 mm Tris‐HCl (pH 7.5), 2 mm EDTA, 1% Triton X‐100); Buffer C (10 mm Tris‐HCl (pH 7.5), 250 mm LiCl, 1 mm EDTA, 1% NP‐40, 0.5% sodium deoxycholate); and Buffer D (10 mm Tris‐HCl (pH 7.5), 1 mm EDTA, 0.1% Triton X‐100). Bound complexes were eluted with elution buffer (1% SDS, 0.1 m NaHCO_3_). After reversing cross‐links overnight with 5 m NaCl at 65°C, DNA was purified via phenol‐chloroform extraction and ethanol precipitation. Precipitated DNA was resuspended in water and analyzed by qPCR using primers specific to the *CathB3* promoter. Results were normalized to input DNA. The primer for CHIP‐qPCR is shown in Table S1.

### 4.18 Transient Expression in Protoplasts and *N. benthamiana*

Arabidopsis mesophyll protoplasts were isolated from leaves of 3–4‐week‐old plants using cellulase and macerozyme enzymes. Protoplasts were transfected with plasmid DNA using PEG‐mediated transformation. For transactivation assays, a reporter plasmid containing the *CathB3* promoter driving LUC and an internal control plasmid expressing REN were co‐transfected with effector plasmids. Luminescence was measured 16–24 h after transfection using the Dual‐Luciferase Reporter Assay System (Promega, E1910). LUC activity was normalized to REN activity.

For transient expression in *N. benthamiana*, Agrobacterium strain GV3101 carrying the desired plasmids was grown, resuspended in infiltration buffer (10 mm MgCl_2_, 10 mm MES (pH 5.7), 200 µm acetosyringone), and infiltrated into leaves of 3–4‐week‐old plants. Tissues were harvested 24–48 h post‐infiltration for analysis.

### 4.19 Fluorescence Imaging of Cells and Tissues

Arabidopsis seed imaging. Arabidopsis seeds were stratified at 4°C for 3 days in the dark and then transferred to a growth chamber under light conditions. After 24 h of imbibition, seeds were carefully dehusked under a stereomicroscope using fine‐tipped forceps. A dehusked seed was placed on a glass slide in a 10 µL droplet of liquid half‐strength MS medium and gently covered with a coverslip. Imaging was performed using a Nikon AXR confocal microscope system equipped with NSPARC detectors and a ×100/1.45 NA oil immersion objective. Due to the high susceptibility of the seed fluorescence to photobleaching, a low‐intensity laser was first used for preview and to identify regions of interest. Subsequently, images were acquired using a medium laser intensity for excitation and high detector gain to optimize the signal‐to‐noise ratio while minimizing photodamage. Autofluorescence was predominantly detected in the cytoplasm, while the GBF1‐GFP signal was nuclear‐localized.

For imaging of the tobacco leaf epidermal cell, a small leaf disc was excised and soaked in liquid half‐strength MS medium prior to imaging. The leaf disc was mounted on a glass slide and imaged immediately using a Nikon A1 HD25 confocal microscope. For imaging of yeast cells, three independent colonies were streaked onto a fresh medium plate and cultured overnight at 30°C. Before imaging, a single colony was resuspended in the appropriate liquid medium. A small droplet of the cell suspension was sprayed onto a glass slide and covered with a coverslip. Imaging was performed using a Nikon A1 HD25 confocal laser microscope and a ×100/1.45 NA oil immersion objective.

For fluorescence detection, GFP was excited at 488 nM, and emission was collected at 500–550 nM; mVenus was excited at 514 nM, and emission was collected at 529–570 nM.

### 4.20 Fluorescence Lifetime Imaging‐Förster Resonanc FLIM‐FRET)

The solubility tag MBP was first removed from the GBF1‐GFP fusion protein by TEV protease cleavage. The donor, GBF1‐GFP (10 µm), was incubated with the acceptor, a TAMRA‐labeled DNA fragment containing the *CathB3* G‐box (100 nm), in 384‐well microscopy plates for 30 min at room temperature to allow complex formation before imaging.

FLIM‐FRET imaging was carried out on a Leica TCS SP8 laser‐scanning confocal microscope equipped with a 100×/1.40 NA oil immersion objective and FLIM capability. The donor fluorescence (mGFP) was excited at 488 nM using a pulsed white light laser, and photon events were recorded using a time‐correlated single photon counting module. To accumulate sufficient photon counts for robust fitting, the selected region of interest (ROI) was repeatedly scanned 30–100 times. Photon arrival times were fitted to a double‐exponential reconvolution model using the Leica LAS X FLIM FCS software, and the mean fluorescence lifetime was calculated based on intensity weighting. A decrease in the donor fluorescence lifetime indicates the occurrence of FRET and thus close proximity between GBF1 and the DNA probe. For each sample, fluorescence lifetimes were analyzed from a minimum of six distinct ROIs.

### 4.21 β‐Galactosidase Liquid Assay

The *Saccharomyces cerevisiae* reporter strain EGY48 was used for all two‐hybrid assays. Positive cotransformants were obtained by co‐transforming the yeast strain with the pLacZi reporter plasmid (*pCathB3‐LacZi*) and the pB42AD fusion plasmid (*pB42AD*, *pB42AD‐GBF1* ^*PrLDm*^, *pB42AD‐GBF1* ^*FUSN*^, and *pB42AD‐GBF1* ^*ΔPrLD*^) using a high‐efficiency LiAc/PEG method. Briefly, yeast competent cells were prepared by washing overnight YPD cultures with sterile dH_2_O and resuspending them in a freshly prepared One‐step buffer (0.1 m LiAc, 40% PEG3350, 0.1% β‐mercaptoethanol). A mixture of 2.5 µL of each plasmid and 5 µL of sheared salmon sperm DNA was added to 100 µL of competent cells. After incubation at 45°C for 30 min with intermittent vortexing, the transformed cells were selected on synthetic dropout (SD)/‐Trp‐Ura solid medium and incubated at 30°C for 2–4 days. β‐Galactosidase activity was quantitatively measured from liquid cultures of these positive cotransformants using the ortho‐Nitrophenyl‐β‐galactoside (ONPG) hydrolysis assay. Single colonies were inoculated into 5 mL of SD/‐Trp‐Ura liquid selection medium and grown overnight at 30°C with shaking (200 rpm). The overnight culture was diluted into 8 mL of YPD medium and incubated until the cells reached mid‐log phase (OD600 = 0.5–0.8). Cells were harvested, washed, and concentrated 5‐fold in Z buffer. A 100 µL aliquot of the cell suspension was permeabilized by three freeze‐thaw cycles using liquid nitrogen and a 37°C water bath. The enzymatic reaction was initiated by adding 700 µL of Z buffer with β‐mercaptoethanol and 160 µL of ONPG substrate (4 mg/mL in Z buffer) and incubating at 30°C. The reaction was terminated by adding 400 µL of 1 m Na_2_CO_3_ upon visible yellow color appearance, and the elapsed time was recorded. After centrifugation to remove cell debris, the absorbance of the supernatant was measured at 420 nM. β‐Galactosidase activity was calculated in Miller Units using the formula:

Activity=1000×OD420/(t×V×OD600)

where “*t*” is the reaction time (min), *V* is the volume of culture assayed (0.1 mL × concentration factor), and *OD600* is the optical density of the original culture. All assays were performed in triplicate.

### 4.22 Yeast One‐Hybrid Assay for Transcriptional Activation of GAL4

The constructed plasmids (*pBKT7*, *pBKT7‐GBF1*, *pBKT7‐ GBF1* ^*PrLDm*^, *pBKT7‐ GBF1* ^*FUSN*^, *pBKT7‐GBF1* ^*ΔPrLD*^) were individually transformed into the *Saccharomyces cerevisiae* reporter strain AH109, which contains integrated GAL4‐responsive reporter genes (*HIS3*, *ADE2*), using the high‐efficiency LiAc/PEG method as previously described. Positive transformants were selected on SD/‐Trp solid medium. For transcriptional activation analysis, three independent colonies of each transformation were spotted onto SD/‐Trp control plates and SD/‐Trp/‐His/‐Ade triple‐dropout selection plates. The plates were incubated at 30°C for 3–5 days, and growth was documented photographically. Activation of the reporter genes was assessed based on colony growth under selective conditions.

### 4.23 Yeast Two‐Hybrid Assay

The constructed plasmids (*pGADT7*, *pGBKT7*, *pGADT7‐GBF1*, *pGBKT7‐GIP1*, *pGBKT7‐GIP1‐4CS*) were co‐transformed into yeast strain AH109, using the high‐efficiency LiAc/PEG method as previously described. Transformants were selected on SD medium lacking Leu and Trp. Protein interactions were assessed by growth on SD medium lacking Leu, Trp, His, and Ade.

### 4.24 Turbidity Measurements

Purified MBP‐GBF1 was treated with TEV protease to remove the MBP tag. The cleaved GBF1 protein was then diluted to a final concentration of 5 µm in phase separation buffer (40 mm Tris‐HCl (pH 7.4), 150 mm NaCl) and incubated with increasing concentrations of GIP1 (0, 1, 3, and 5 µm). The mixtures were transferred to a flat‐bottom 96‐well plate (Corning, 3364), and turbidity was measured at 600 nM using a Varioskan Flash microplate reader (Thermo Scientific). All measurements were performed in triplicate.

### 4.25 Redox Treatment and Electrophoretic Analysis of GIP1

Wild‐type GIP1 was incubated in either a reducing buffer (5 mm DTT, 500 mm NaCl, 20 mm Tris‐HCl (pH 7.4)) or an oxidizing buffer (3 mm H_2_O_2_, 500 mm NaCl, 20 mm Tris‐HCl (pH 7.4)) for 1 h at room temperature. To remove DTT or H_2_O_2_ while maintaining the protein in its reduced or oxidized state, the samples were subjected to buffer exchange using centrifugal concentrators, with three to four washes in a storage buffer (500 mm NaCl, 20 mm Tris‐HCl (pH 7.4)). The redox states of the treated proteins were then analyzed by non‐reducing 8% SDS‐PAGE. Samples for non‐reducing conditions were prepared in loading buffer without β‐mercaptoethanol, while a control sample was prepared in loading buffer containing β‐mercaptoethanol to represent the fully reduced form.

### 4.26 Statistical Analysis

Data are presented as mean ± SD or SEM from at least three independent biological replicates, as indicated in the figure legends. For comparisons between two groups, statistical significance was determined using an unpaired two‐tailed Student's *t*‐test, with significance levels indicated by asterisks (^***^ *p* < 0.001, ^**^ *p* < 0.01, ^*^ *p* < 0.05). For comparisons involving more than two groups, one‐way ANOVA followed by Tukey's post‐hoc test was used, and groups with different letters indicate significant differences (*p* < 0.05). Statistical analyses were performed using IBM SPSS Statistics or GraphPad Prism software.

## Author Contributions

X.F. conceived, guided, and supervised the project. Y.W. performed all experiments. X.F. and Y.W. wrote the manuscript together.

## Conflicts of Interest

The authors declare no conflicts of interest.

## Supporting information




**Supporting File 1**: advs75831‐sup‐0001‐SuppMat.docx.

**Supporting File 2**: advs75831‐sup‐0002‐Supplementary Table 1.xlsx.

## Data Availability

The data that support the findings of this study are available in the supplementary material of this article.
