# Supplementary material for: Redox‐Dependent Chaperoning of GBF1 Condensates Regulates Seed Germination in Arabidopsis
Source: Adv Sci (Weinh). 2026 May 26:e20599. Online ahead of print. doi: 10.1002/advs.202520599 (PMC13335883; doi:10.1002/advs.202520599)
Supplement: Supplementary file 1 — Supporting File 1: advs75831‐sup‐0001‐SuppMat.docx. [file ADVS-9999-e20599-s001.docx]

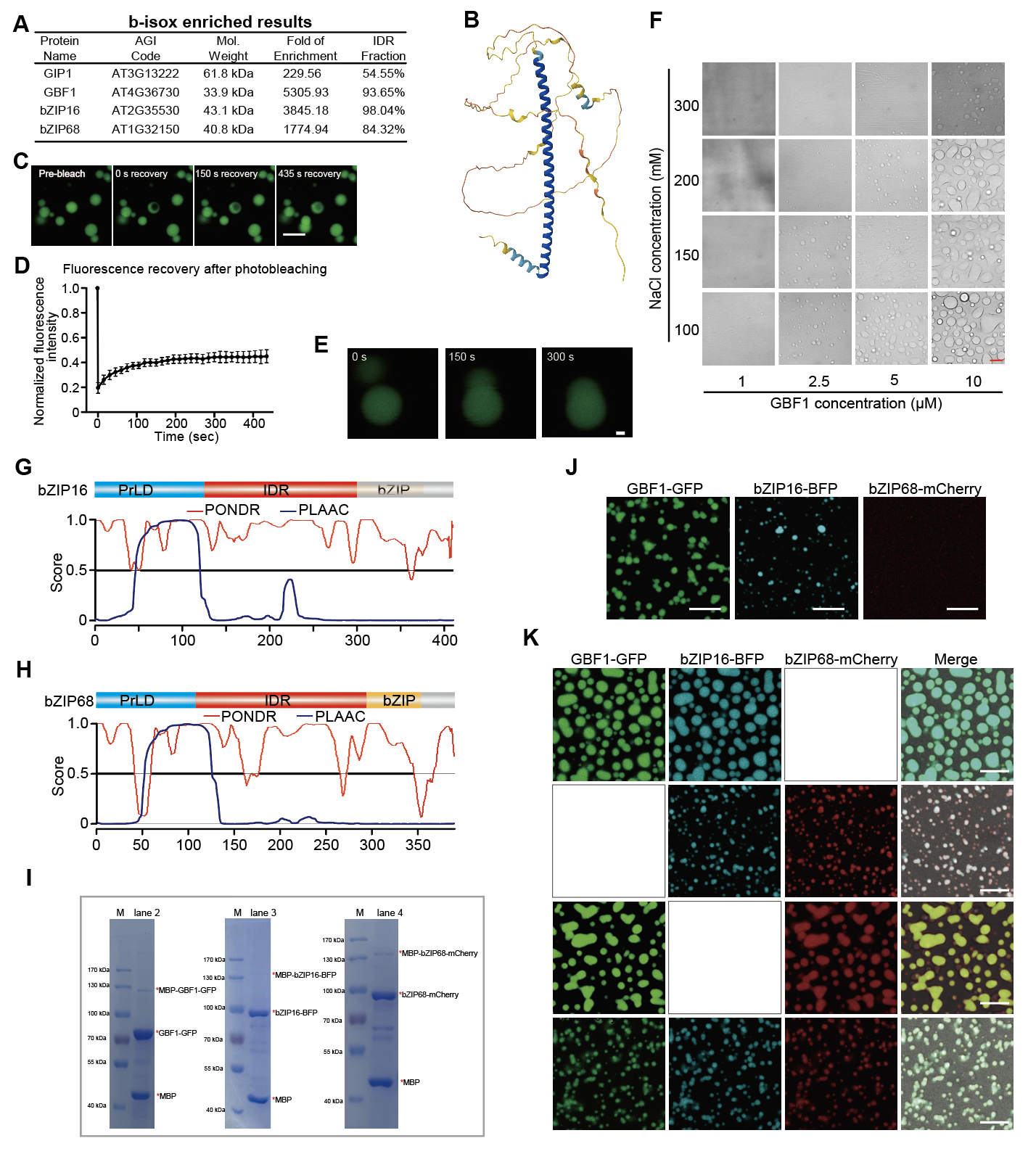


**Figure S1 | Characterization of the phase separation of GBF1, bZIP16, and bZIP68.**

1. A table showing the enrichment of GIP1, GBF1 and two bZIP proteins by b-isox precipitation. B) Alphafold2 prediction of GBF1 structure. C) FRAP of GBF1-GFP condensates. Time 0 sec indicates the time of the photobleaching pulse. Scale bar, 5 µm. D) FRAP recovery curve. E) Confocal microscopy showing the fusion of two GBF1-GFP droplets. Scale bar, 1 µm. F) In vitro phase diagram of untagged GBF1 protein. Scale bar, 10 µm. G-H) Prediction of the intrinsically disordered regions (IDRs) and prion-like domain (PrLDs) of bZIP16 and bZIP68 by PONDR and PLACC algorithms, respectively. I) Coomassie blue staining of the purified MBP-GBF1-GFP, MBP-bZIP16-BFP, and MBP-bZIP68-mCherry proteins after TEV cleavage to release the MBP. Lane M: molecular weight marker. J) In vitro phase separation assay for GBF1, bZIP16 or bZIP68. Scale bars, 10 µm. K) In vitro phase separation assays for the indicated combinations of proteins. Scale bars, 10 µm.


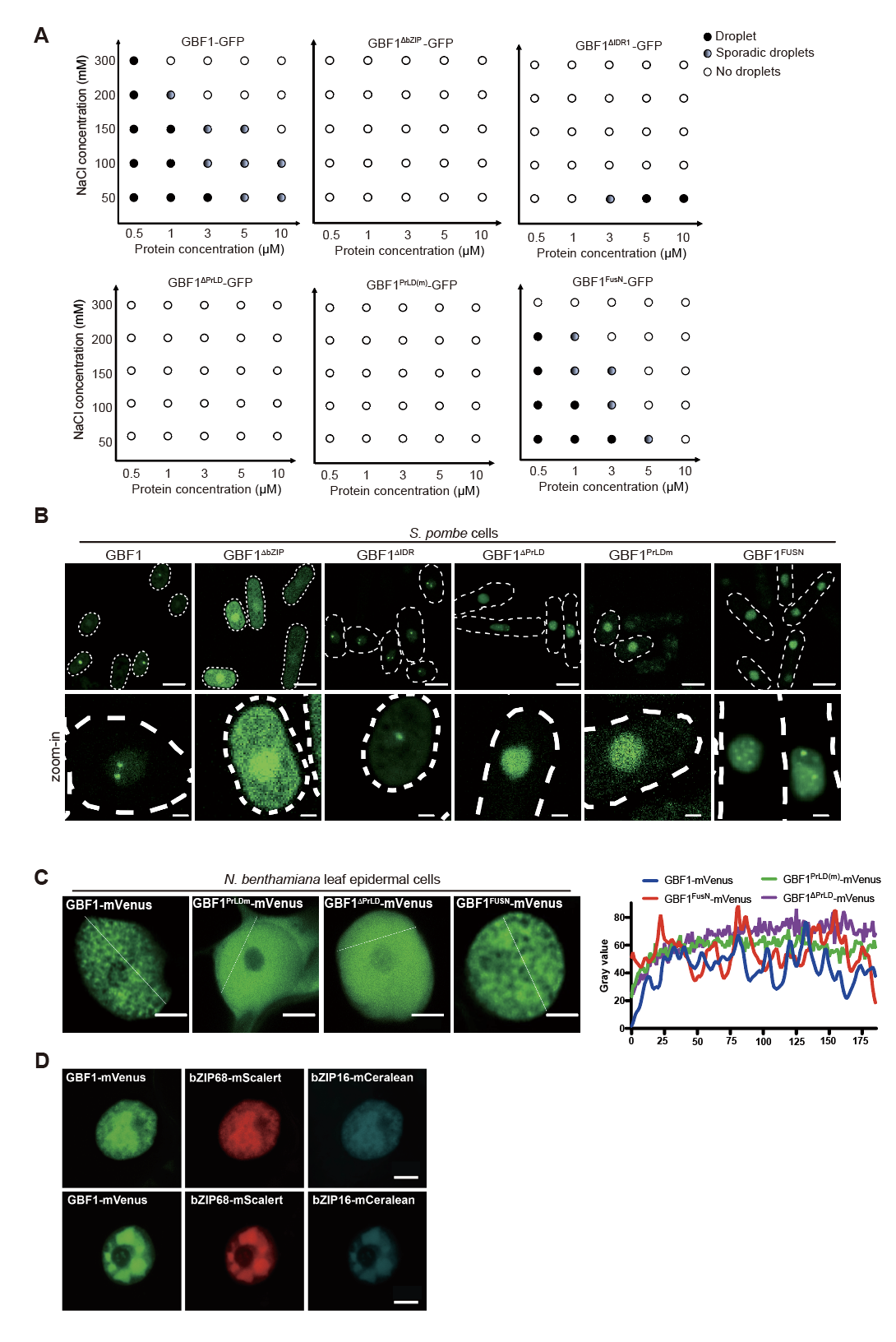
 **Figure S2 | Analysis of GBF1 condensation in vitro and in vivo.**

A) Phase diagrams of GBF1 and its variants in vitro. Purified proteins (GBF1, GBF1^ΔPrLD^, GBF1^ΔIDR^, GBF1^ΔbZIP^, GBF1^PrLDm^, and GBF1^FUSN^) were diluted to indicated concentrations (0.5-10 µM) in buffers containing increasing concentrations of NaCl (50-300 mM). Condensate formation was assessed by confocal microscopy after 30 min incubation at room temperature. B) Representative confocal images of *Schizosaccharomyces pombe* cells expressing GBF1-GFP or its variants. Top scale bars, 10 µm. Bottom scale bars, 2 µm. C) Left, representative confocal images of tobacco epidermal cells expressing GBF1-GFP or its variants. Scale bars, 5 µm. Right, fluorescence plots of the white line shown in left. D) Colocalization of bZIP16-mCerulean and bZIP68-mScarlet with GBF1-mVenus in *N. benthamiana* epidermal cells. Scale bars, 5 µm.


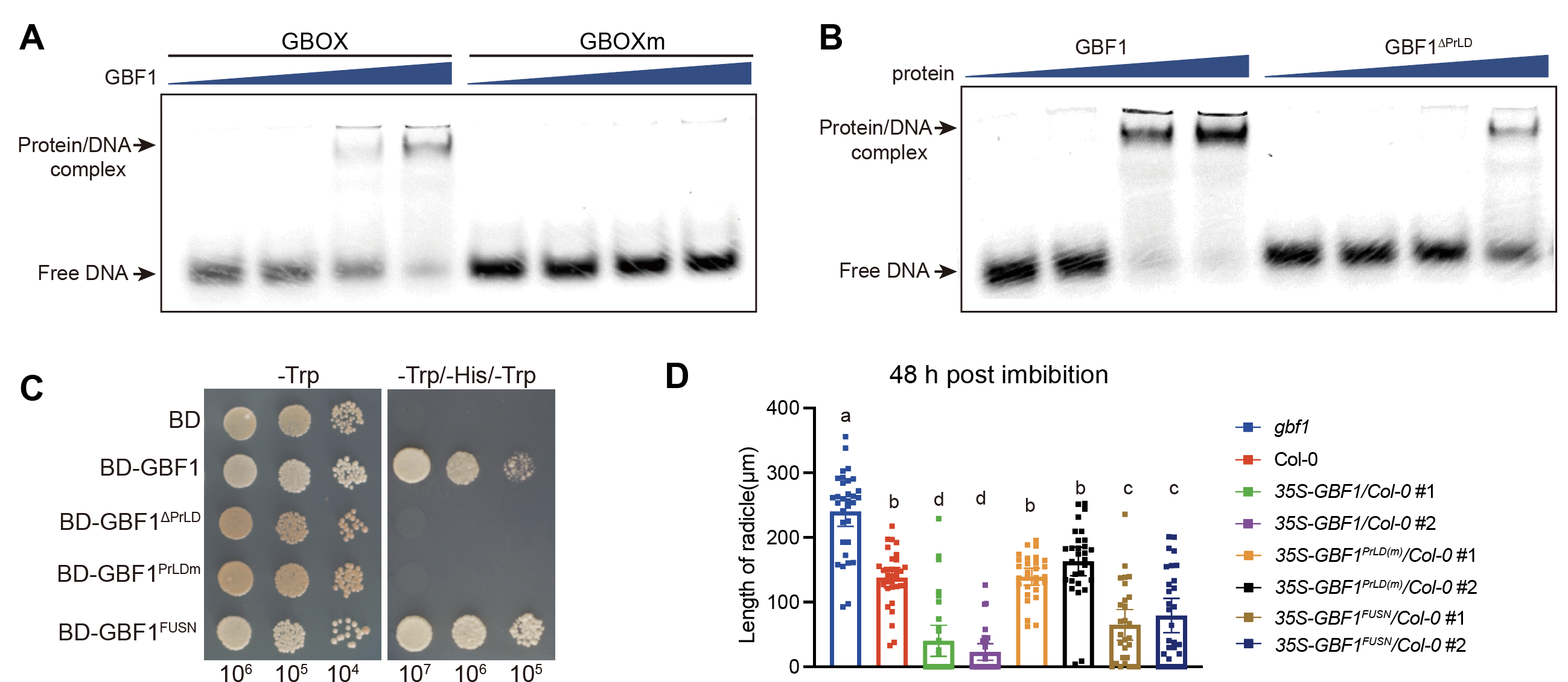
 **Figure S3 | DNA binding and self-activation of GBF1.**

A) EMSA showing specific binding of GBF1 to the GBOX probe, but not to GBOXm probe. B) EMSA showing the binding of wild-type GBF1 and the phase separation-deficient GBF1^∆PrLD^ variant to GBOX probe. C) Yeast one-hybrid self-activation assay for the indicated GBF1-DBD (DNA-Binding Domain) fusion constructs. D) Radicle lengths of the indicated transgenic lines at 48 hours post imbibition (HAI). Data are mean ± SD (n≥28). Different letters above the bars indicate statistically significant differences (*P* < 0.05, one-way ANOVA with Tukey's post-hoc test).

**
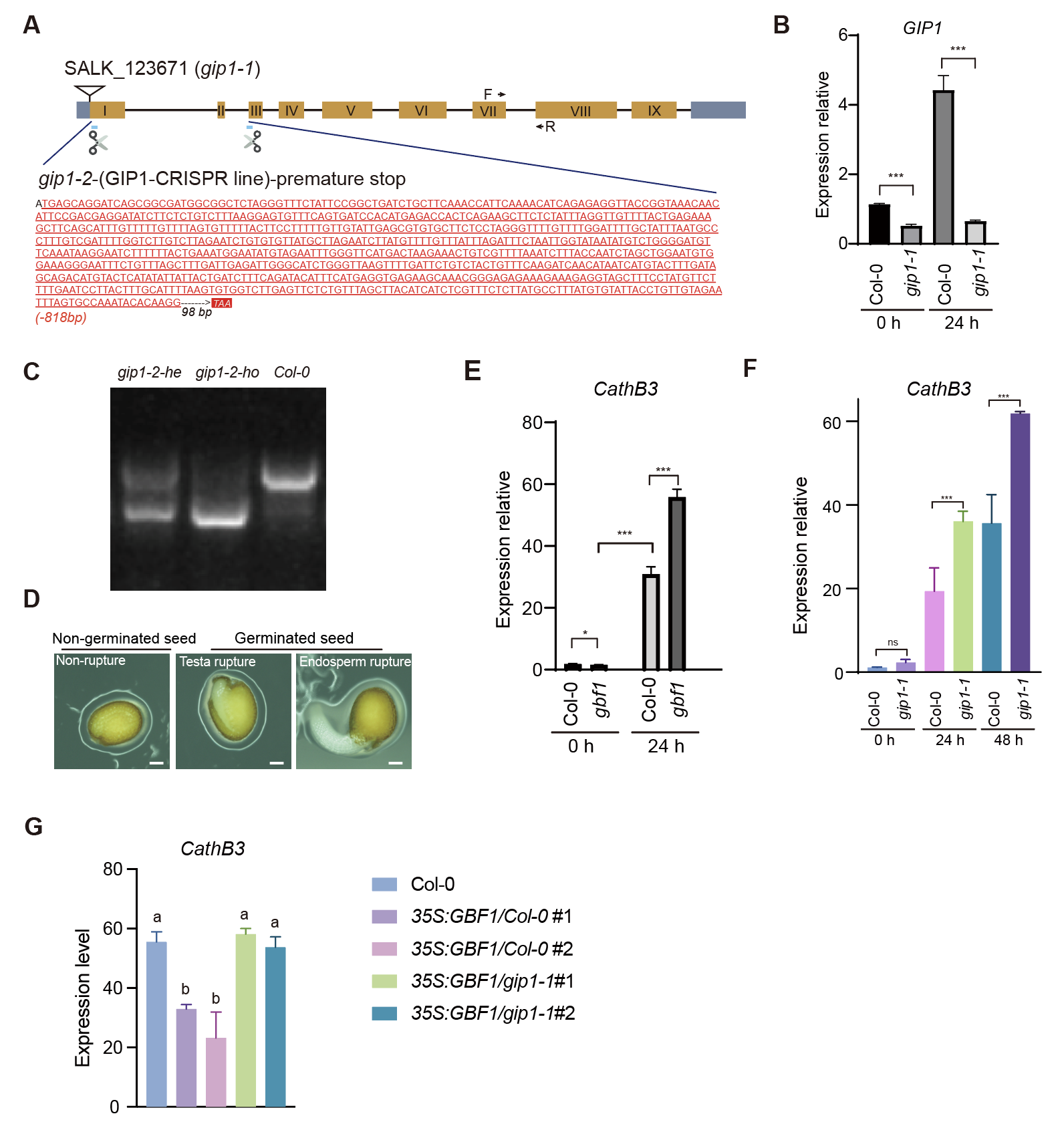
Figure S4 | Analysis of GIP1.**

A) Schematic of the *GIP1* gene structure. The T-DNA insertion line *gip1-1* mutant and CRISPR-Cas9 edited line *gip1-2* mutant were indicated. The deleted 818 bp fragment is highlighted in red. The sequence below shows a premature termination codon (red box) appearing 98 bp downstream of the deletion. B) Quantitative RT-PCR analysis of *GIP1* expression in wild-type (Col-0) and the *gip1-1* mutant seeds. C) Agarose gel electrophoresis of genomic PCR products from *gip1-2* heterozygote (*gip1-2-he*), homozygote (*gip1-2-ho*), and Col-0. D) Representative images of germinating seed (testa rupture and endosperm rupture). Scale bars, 500 µm. (E-F) Quantitative RT-PCR analysis of *CathB3* expression in dry seeds (0 HAI) and during imbibition (24 HAI, 48 HAI) in *gbf1* (E) and *gip1-1* (F) mutants compared to Col-0. Data (in B, E and F) are mean ± SD (n = 3). ****P < 0.001, **P<0.01, *P < 0.05* (unpaired two‑tailed Student’s t‑test). ns, not significant. (G) Relative mRNA expression levels of *CathB3* in the corresponding lines at 48 HAI, determined by qRT-PCR and normalized to *UBC21*. Data are presented as mean ± SD (n = 3). Groups with different letters indicate significant differences (*P* < 0.05, one-way ANOVA with Tukey's post-hoc test).


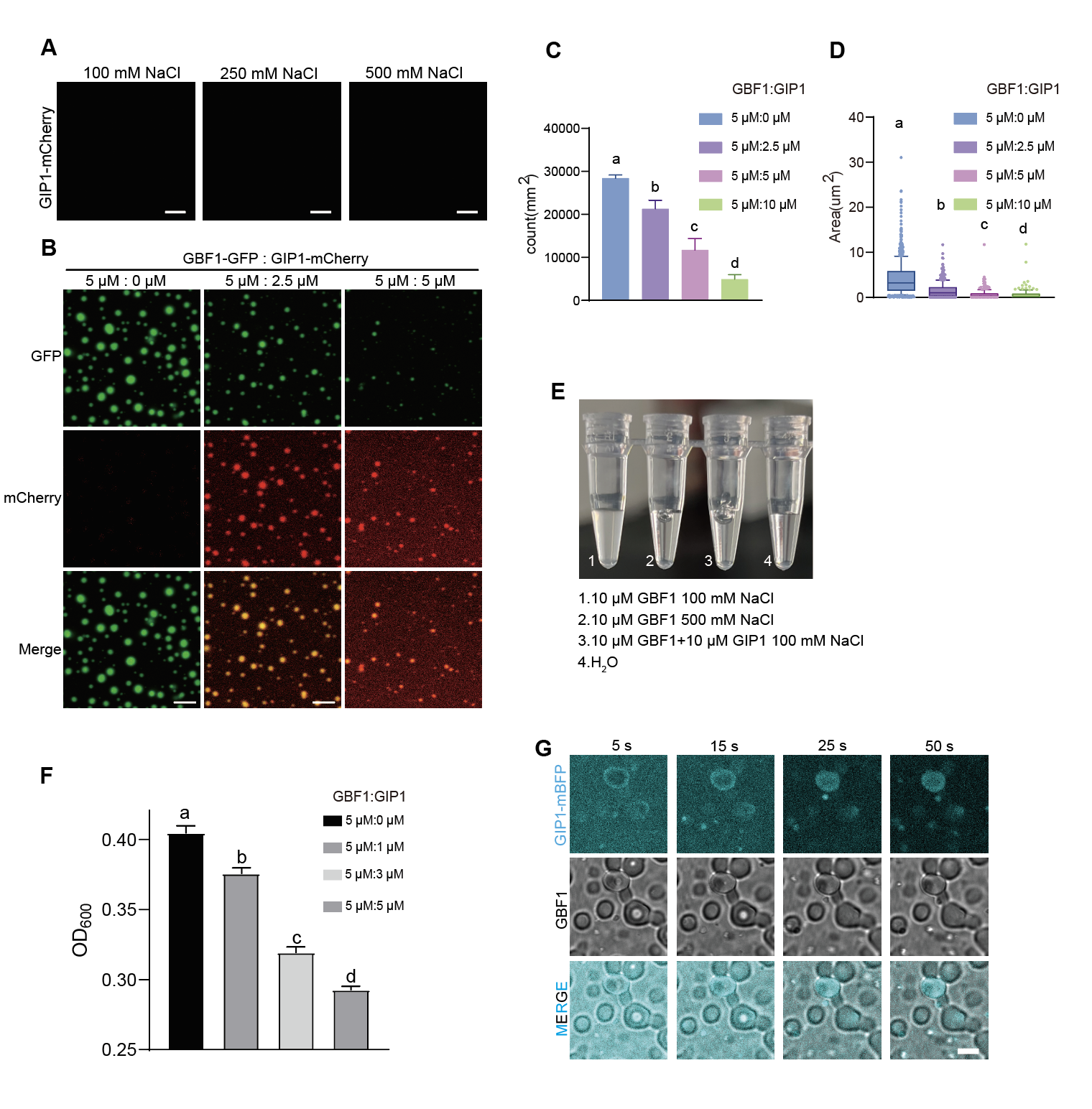
 **Figure S5 | GIP1 modulates GBF1 condensation in vitro.**

A) GIP1-mCherry alone does not form condensates in vitro. Scale bars, 10 µm. B) GIP1-mCherry reduces the size and number of GBF1-GFP condensates in vitro. Scale bars, 10 µm. C) Quantification of the number of GBF1 (5 µM) condensates with increasing concentrations of GIP1 (0-10 µM) in vitro. Data are mean ± SD (n=3). Groups with different letters indicate significant differences (ANOVA, P < 0.05). D) Quantification of the area of GBF1 (5 µM) condensates with increasing concentrations of GIP1 (0-10 µM) in vitro. Data (n ≥ 142) are presented as box plots showing the mean (center line), 25th/75th percentiles (box), and data range (whiskers). Groups with different letters indicate significant differences (ANOVA, P < 0.05). E) Image showing the solution turbidity of the indicated protein samples. F) Turbidity measurements of TEV-cleaved GBF1 (5 μM) incubated with increasing concentrations of GIP1 (0, 1, 3, 5 μM) in 40 mM Tris-HCl (pH 7.4) and 150 mM NaCl. Data are mean ± SD (n=3). Groups with different letters indicate significant differences (*P* < 0.05, one-way ANOVA with Tukey's post-hoc test). G) Time-lapse imaging showing the distribution of GIP1 from GBF1 condensate speriphery to interior. Scale bar, 5 µm.


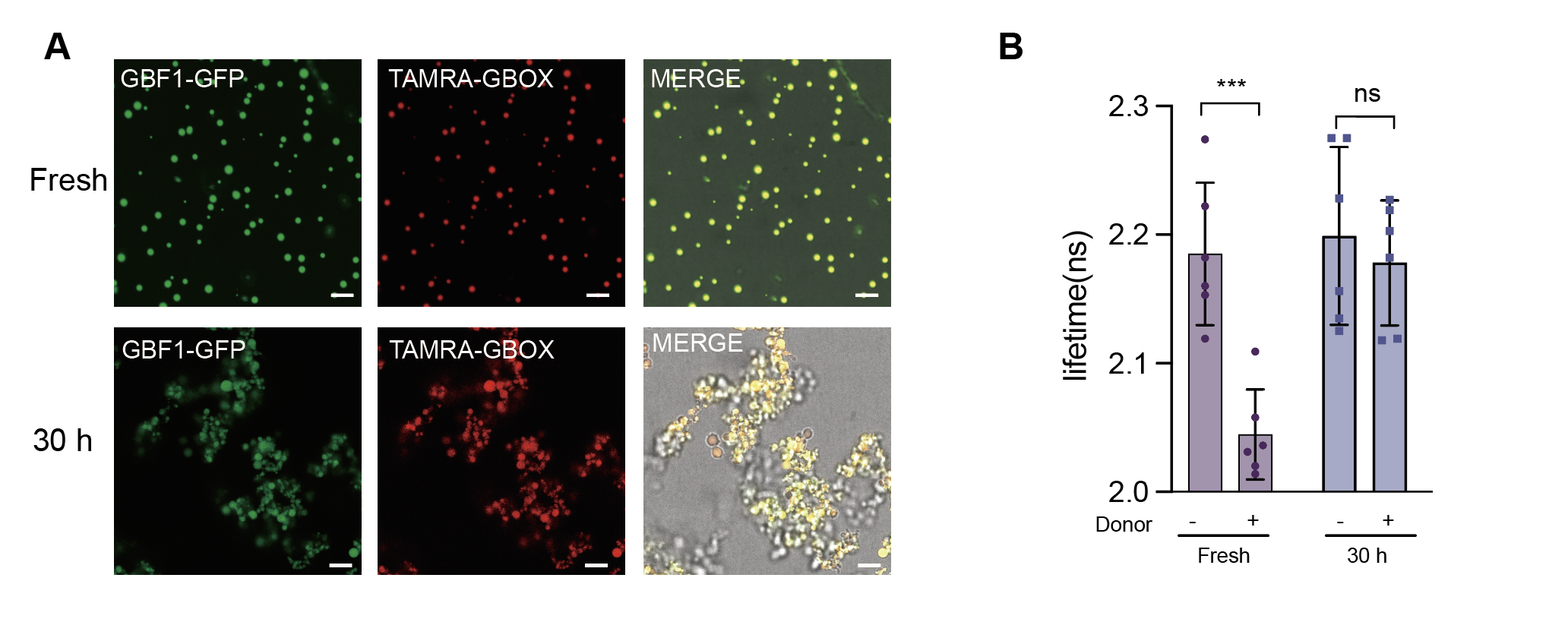


**Figure S6 | FLIM‑FRET analysis of DNA binding to liquid versus solid/aged GBF1 condensates.**

A) Representative confocal images of freshly prepared (liquid) GBF1 condensates and aged (30 h on ice, solid) GBF1 condensates after incubation with a TAMRA‑labeled *CathB3* promoter DNA probe. GBF1 is tagged with GFP (green); DNA probe is shown in red. Scale bars, 10 μm. B) Quantification of donor (GFP) fluorescence lifetime measured within GBF1 condensates using Leica SP8 in the absence (‑DNA) or presence (+DNA) of the TAMRA-labeled *CathB3* probe. Data represent mean ± SD from six independent condensates per condition. Statistical significance was determined by unpaired two‑tailed Student’s t‑test (****P < 0.001, **P<0.01, *P < 0.05*; ns, not significant).


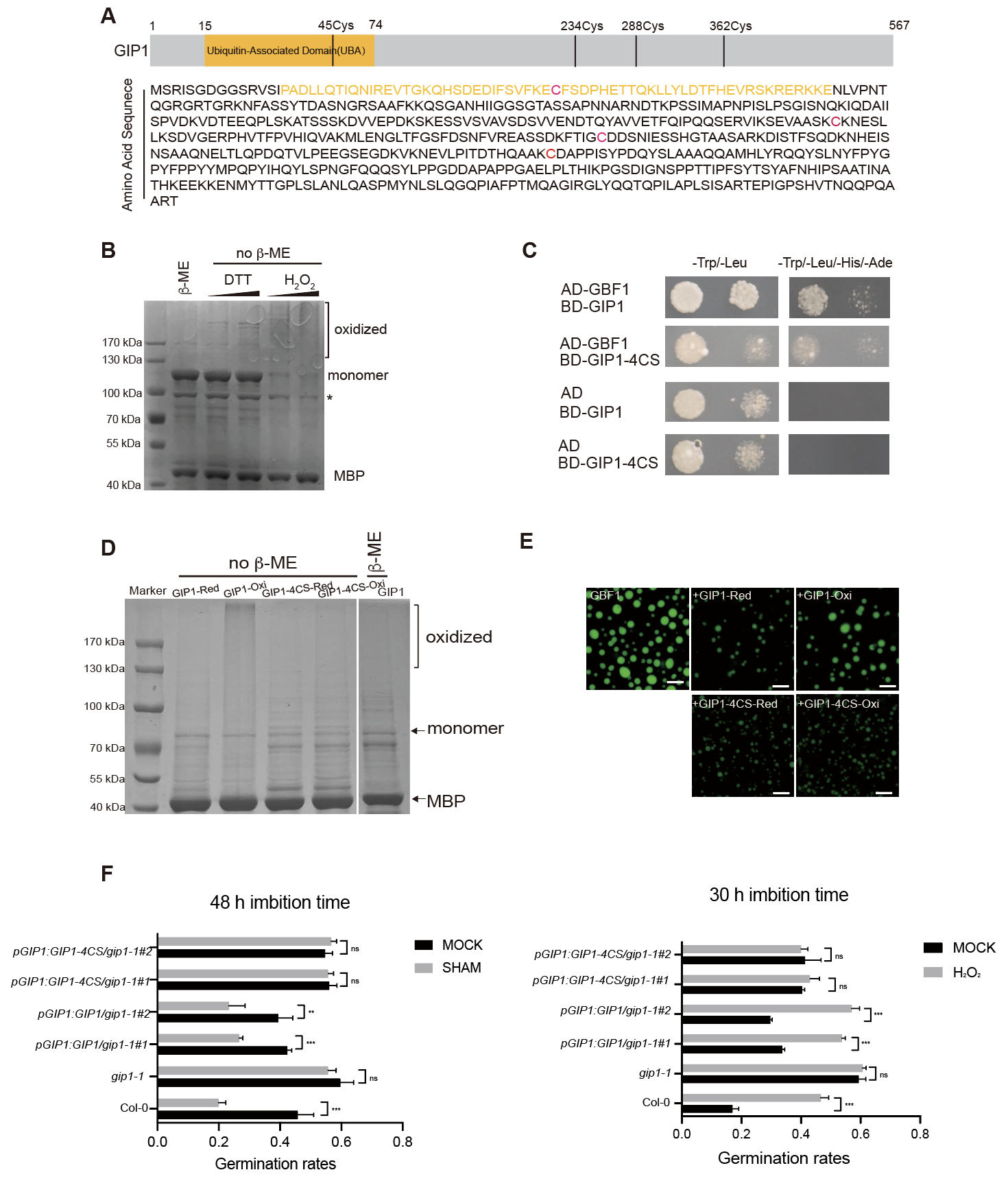
 **Figure S7 | The activity of GIP1 is sensitive to redox.**

A) Protein sequence of GIP1 highlighting the four cysteine residues (red). B) Redox-dependent oligomerization of GIP1 as analyzed by non-reduced SDS-PAGE. Purified GIP1 protein was treated with the DTT (1 mM, 3 mM) or H_2_O_2_ (1 mM, 3 mM) and analyzed by SDS-PAGE under non-reduced conditions (without β-mercaptoethanol). The positions of monomeric and oligomeric forms of GIP1, as well as the MBP tag were indicated. C) Yeast two-hybrid assay detecting the interaction of wild-type GIP1 or GIP1-4CS with GBF1. D) Non-reducing SDS-PAGE analysis of the redox states of wild-type GIP1 and GIP1-4CS. Lane M: protein marker; lane 2: GIP1-Red; lane 3: GIP1-Oxi; lane 4: GIP1-4CS-Red; lane 5: GIP1-4CS-Oxi; lane 6: fully reduced GIP1 control (with β-mercaptoethanol). E) *In vitro* imaging showing the effects of GIP1 and GIP1-4CS under different redox conditions on GBF1 condensates, which were incubated in buffer (40 mM Tris-HCl, pH 7.4, 150 mM NaCl). Scale bars, 10 μm. F) Seeds were germinated on 1/2 MS medium supplemented with 400 µM SHAM or pretreated with 25 mM H_2_O_2_. Germination (radicle emergence) was scored at 48 hours of imbibition for SHAM treatment and at 30 hours for H_2_O_2_ treatment. Data are mean ± SEM (n = 3 independent replicates; ≥80 seeds per replicate). ****P* < 0.001, ***P* < 0.01, **P* < 0.05 (unpaired two‑tailed Student’s t‑test). ns, not significant.

**Materials and Methods**

**Plant materials and growth conditions**

All the Arabidopsis thaliana mutants and transgenic plants used in this study were in the Columbia (Col-0) background. The T-DNA insertion mutants *gip1-1* (SALK_123671, insertion in the 5′ UTR), *cathb3* (WiscDsLox336C03, insertion in the 5′ UTR) were obtained from the AraShare and *gbf1* (SALK_144534, insertion in the exon 5) were obtained from the Arabidopsis Biological Resource Center (ABRC), respectively. The *gip1-1 gbf1, gip1-1 cathb3 and gbf1 cathb3* double mutant were generated by genetic crossing of the respective single mutants. To generate the *gip1-2* CRISPR allele, two sgRNAs targeting exon 1 and exon 3 of *GIP1* were designed and cloned into a CRISPR-Cas9 vector. Transgenic plants were selected, and the 818 bp deletion was confirmed by PCR and Sanger sequencing. Two sgRNAs targeting sequence are listed in Supplementary Table S1.

Seeds were surface-sterilized with 75% ethanol for 5 min, followed by three washes with sterile water, and then treated with 10% sodium hypochlorite containing 0.1% Tween-20 for 3-5 min. After five additional washes with sterile water, seeds were sown on half-strength Murashige and Skoog (1/2 MS) medium containing 0.8% (w/v) agar and 1% (w/v) sucrose. Plates were stratified at 4°C in the dark for 3 days and subsequently transferred to a growth chamber. Plants were grown under long-day conditions (16 h light / 8 h dark) at 20-22°C with a light intensity of 100-150 µmol m^-2^s^-1^. Plate media were transferred to a growth chamber after about 7 days. *Nicotiana benthamiana* plants were grown in soil under similar conditions for 3-4 weeks before using in transient expression assays.

**Germination Phenotype Analysis**

To ensure uniformity in seed physiological status, all seeds used in germination assays were from plants grown and harvested simultaneously, followed by a three-month after-ripening period under dry, ambient conditions to break dormancy. Mature, after-ripened seeds from different genotypes were sown on half-strength Murashige and Skoog (1/2 MS) agar plates. Plates were stratified at 4°C in the dark for 3 days and subsequently transferred to a growth chamber under standard conditions. Germination, defined as radicle emergence through the seed coat, was scored every 12 hours. For each experiment, a minimum of three biological replicates were performed, with each replicate consisting of 80-100 seeds per genotype and condition.

For reactive oxygen species (ROS) manipulation assays, seeds were germinated on plates supplemented with 400 µM salicylhydroxamic acid (SHAM), a peroxidase inhibitor. Alternatively, to assess the effect of exogenous H₂O₂, seeds of different genotypes were soaked overnight in an aqueous solution of 25 mM H₂O₂ prepared in liquid 1/2 MS medium, and kept in the dark. The following morning, the treated seeds were blotted dry and sown on standard 1/2 MS agar plates for germination scoring. For each experiment, a minimum of three biological replicates were performed, with each replicate consisting of 80-100 seeds per genotype and condition.

**Genotyping**

Genomic DNA was extracted from leaf tissue. T-DNA insertion lines were genotyped by PCR using gene-specific primers in combination with T-DNA left border primer LBb1.3. Genotyping of *gip1-2* was performed using primers, yielding a wild-type band of about 3K bp and a deletion band of about 2K bp. Primer sequences used for genotyping are listed in Supplementary Table S1. PCR products were analyzed by agarose gel electrophoresis.

**Molecular Cloning**

The coding sequences (CDS) of GIP1, bZIP16, bZIP68 and GBF1 (and their variants) were amplified from Arabidopsis cDNA using Phusion High-Fidelity DNA Polymerase (Thermo Fisher Scientific) or Platinum SuperFi II DNA Polymerase (Thermo Fisher Scientific). Amplified fragments were cloned into appropriate destination vectors (e.g., pCAMBIA1300 for plant transformation, pET-based vectors for bacterial expression, pDUAL for yeast expression, pB42AD and pLacZi for β-Galactosidase Liquid Assay, pADT7 and pBKT7 for yeast one- or two-hybrid assay) using either restriction enzyme-based cloning or the ClonExpress Ultra One Step Cloning Kit (Vazyme, C115). Site-directed mutagenesis (e.g., GIP1-4CS, GBF1^PrLDm^) was performed using a standard two-step PCR method or Chemical synthesis. All constructs were verified by Sanger sequencing. Primer sequences are available in Supplementary Table S1.

**Plasmid construct**

The *pGBF1::GBF1-mVenus* construct was generated by amplifying a 1.8 kb promoter region upstream of the *GBF1* start codon from wild-type Col-0 genomic DNA and cloning it into the pCAMBIA1300-mVenus vector, resulting in an intermediate *pGBF1::mVenus* vector. The coding sequence (CDS) of *GBF1* was then inserted between the promoter and the *mVenus* tag. Using the same vector backbone and a similar strategy, we also generated the *pGIP1::GIP1-mVenus* construct, in which the *GIP1* CDS was placed under the control of its 1.6kb promoter. Additionally, a redox-insensitive version, *pGIP1::GIP1-4CS-mVenus*, was created by introducing point mutations (cysteine-to-serine substitutions at four conserved residues) via site-directed mutagenesis and overlap extension PCR.

To generate the constructs used for in vitro protein expression, the following procedures were employed in pET-based vectors. The coding sequences for *GBF1^ΔPrLD^*, *GBF1^ΔIDR^*, and *GBF1^ΔbZIP^* were generated using a standard two-step PCR method to remove the respective domains (prion-like domain, intrinsically disordered region, or basic leucine zipper domain) and verified by DNA sequencing. To generate the *GBF1^FUSN^* variant, the *FUSN* domain was cloned and fused to the *GBF1^ΔPrLD^* CDS via overlap extension PCR. Similarly, the *GBF1^PrLDm^* variant was produced by synthesizing a mutated *PrLD* sequence (phenylalanine and tyrosine-to-serine substitutions at 11 conserved residues) and fusing it to the *GBF1^ΔPrLD^* CDS using overlap extension PCR. The coding sequences of GBF1 and its variants (*GBF1^ΔIDR^*, *GBF1^ΔbZIP^*, *GBF1^ΔPrLD^*, *GBF1^PrLDm^*, and *GBF1^FUSN^*), GIP1 and its variants (GIP-4CS), were amplified and inserted into the pET11-6×His-GFP, pET11-6×His-mCherry expression vector (digested with BamHI). To generate the in vitro purified GBF1 without GFP tag, pET11-6×His-GFP was digested by BamHI and XhoI to insert GBF1 CDS. Where necessary, a maltose-binding protein (MBP) solubility tag was placed at the N-terminus of the construct, followed by a tobacco etch virus protease (TEV) cleavage site.

For plant transformation, GBF1, *GBF1^ΔPrLD^*, *GBF1^PrLDm^*, and *GBF1^FUSN^* fragment were subsequently inserted between the *35S* promoter and the *mVenus* coding sequence in the pCAMBIA1300-35S-mVenus vector via homologous recombination, yielding the respective construct (*35S::GBF1-mVenus*, *35S::GBF1^ΔPrLD^-mVenus*, *35S::GBF1^PrLDm^-mVenus*, and *35S::GBF1^FUSN^-mVenus*). Using the same vector backbone and a similar strategy, we also generated the *35S::GIP1-mVenus* construct, in which the GIP1 CDS was placed under the control of 35S promoter.

To generate the constructs used for heterologous expression in yeast cells, the coding sequences of GBF1 and its variants (*GBF1^ΔIDR^*, *GBF1^ΔbZIP^*, *GBF1^ΔPrLD^*, *GBF1^PrLDm^*, and *GBF1^FUSN^*) were amplified and inserted into the pDUAL-Pnmt1-yeGFP vector (digested with NheI and BamHI).

To generate fluorescently tagged constructs for protein localization in *N. benthamiana* epidermal cells, the coding sequences (CDS) of *bZIP16* and *bZIP68* were individually cloned into plant expression vectors under the control of the *Cauliflower Mosaic Virus* (CaMV) *35S* promoter. Specifically, the *bZIP16* CDS was inserted into the *pCAMBIA1300-35S-mCerulean* vector using the SalI restriction site, creating *35S::bZIP16-mCerulean* construct. Similarly, the *bZIP68* CDS was cloned into the pCAMBIA1300-35S-mScarlet-I vector using the BamHI site, resulting in the *35S::bZIP68-mScarlet-I* construct.

To analyze the transcriptional repression activity of the GBF1, constructs for transexpression in protoplasts were produced. A 1.1 kb genomic fragment spanning from -1162 bp to the translation start site (ATG) was amplified and inserted into the pGreenII 0800-LUC vector via the BamHI and SpeI restriction sites, resulting in the reporter construct *pCathB3::LUC*. This plasmid contains a firefly luciferase (LUC) gene driven by the *CathB3* promoter and an internal control Renilla luciferase (REN) gene under a constitutive promoter. For effector constructs, the coding sequences (CDS) of GBF1, GBF1^PrLDm^ and GIP1 were amplified and inserted downstream of the Cauliflower Mosaic Virus (CaMV) 35S promoter in the pGreenII 62-SK vector using the SpeI and BamHI restriction sites. This resulted in the generation of the *35S::GBF1, 35S:: GBF1^PrLDm^* and *35S::GIP1* overexpression constructs, respectively. These constructs were subsequently used in transient transfection assays of Arabidopsis mesophyll protoplasts.

For β-galactosidase activity assay, the coding sequences of *GBF1* and its variants (GBF1^PrLDm^, GBF1^FUSN^, and GBF1^ΔPrLD^) were amplified and inserted into the pB42AD vector using the pBKT7 and XhoI restriction sites, resulting in in-frame fusions downstream of the B42 transcriptional activation domain. For the reporter construct, a 1.1-kb DNA fragment of the *CathB3* promoter was cloned into the pLacZi vector via the KpnI and XhoI sites, placing it upstream of the *lacZ* reporter gene.

To assess the transcriptional activation capacity of GBF1 and its phase separation-related variants, a yeast one-hybrid assay was employed. The coding sequences of full-length and mutant versions of GBF1—including GBF1, GBF1^PrLDm^, GBF1^FUSN^, and GBF1^ΔPrLD^—were amplified and inserted downstream of the GAL4 DNA-binding domain (BD) coding sequence in the pGBKT7 vector using the EcoRI and BamHI restriction sites. This resulted in the generation of in-frame GAL4 BD-GBF1 fusion constructs (*pBKT7-GBF1*, *pBKT7-GBF1^PrLDm^*, *pBKT7-GBF1^FUSN^,* *pBKT7-GBF1^ΔPrLD^*).

To explore protein-protein interactions, the coding sequence of *GIP1* was amplified and inserted into the pGADT7 vector using the EcoRI and BamHI restriction sites, creating a fusion with the GAL4 activation domain (AD). Similarly, the coding sequence of *GBF1* was cloned into the pGBKT7 vector using the same restriction sites, generating a fusion with the GAL4 DNA-binding domain (BD).

**Plant transformation**

Binary vectors were introduced into Agrobacterium tumefaciens strain GV3101. Arabidopsis plants (Col-0, *gbf1*, *gip1-1*） were transformed using the floral dip method. Transgenic T1 plants were selected on 1/2 MS plates containing 30 mg/L hygromycin (AMRESCO, K547). Homozygous T3 or T4 lines were used for experiments.

**b‑isox precipitation and mass spectrometry analysis**The b-isox precipitation and mass spectrometry data shown in Figure S1A were derived from our previously published dataset^[19]^. The detailed experimental procedures can be found in that reference.

**RNA Extraction and Quantitative RT-PCR (qRT-PCR)**

Total RNA was extracted from seeds or seedlings using TRIzol reagent (Invitrogen, 15596018) or the TIANGEN Plant RNA Kit (DP452), following the manufacturers' protocols. Contaminating genomic DNA was removed using DNase I (Promega, M6101). First-strand cDNA was synthesized from 1-3 µg of total RNA using M-MLV Reverse Transcriptase (Invitrogen, 28025013) with oligo(dT) and random hexamer primers. qRT-PCR was performed on a QuantStudio 1 Real-Time PCR System (Applied Biosystems) using M5 HiPer SYBR Premix EsTaq (Mei5 Biotechnology). The *UBC21* (At5g25760) gene was used as an internal control for normalization. Gene expression levels were calculated using the 2^(-ΔCt) method. Primer sequences used for qRT-PCR are listed in Supplementary Table S1.

**ROS Detection**

Seed ROS levels were detected using the fluorescent probe 2',7'-dichlorodihydrofluorescein diacetate (H_2_DCFDA). Seeds were peeled and incubated in 10 µM H_2_DCFDA (dissolved in PBS from a 10 mM DMSO stock) for 15-30 minutes in the dark. After washing with PBS, fluorescence was observed and imaged using a fluorescence microscope (excitation/emission ~502/529 nm).

**GUS Staining**

Histochemical GUS staining was performed on tissues from *pGIP1*::GUS transgenic plants using a GUS staining kit (Huayueyang, GT0391) according to the manufacturer's instructions. Tissues were incubated in GUS staining solution at 37°C overnight in the dark. Chlorophyll was cleared using 70-75% ethanol at 65°C before observation and imaging.

**Protein Expression and Purification**

Recombinant proteins were expressed in Escherichia coli Rosetta (DE3) cells. Cultures were grown at 37 °C to an OD600 of 0.6-0.8, and protein expression was induced with 0.4-0.5 mM isopropyl-β-D-1-thiogalactopyranoside (IPTG) at 16-18 °C overnight. Cells were harvested by centrifugation at 4,000 rpm for 15-20 min at 4°C, resuspended in lysis buffer (40 mM Tris-HCl, pH 7.4, 1000 mM NaCl, 10% glycerol), and lysed by sonication. The lysate was clarified by centrifugation. The supernatant was incubated with Ni-NTA beads 6FF (Smart-lifesciences, Ni NTA Beads 6FF: SA00501L) pre-equilibrated with lysis buffer. To minimize nucleic acid contamination, the resin was first washed extensively with a high-salt buffer (40 mM Tris-HCl, pH 7.4, 1 M NaCl, 20-30 mM imidazole, 10% glycerol), followed by washing with standard wash buffer (40 mM Tris-HCl, pH 7.4, 500 mM NaCl, 20-30 mM imidazole). His-tagged proteins were subsequently eluted with elution buffer (40 mM Tris-HCl, pH 7.4, 500 mM NaCl, 250 mM imidazole,). Eluted proteins were further purified by gel filtration chromatography (Superdex-200; GE Healthcare) in storage buffer (40 mM Tris-HCl, pH 7.4, 500 mM NaCl, 1 mM DTT). Protein concentrations were determined spectrophotometrically (NanoDrop One, Thermo Fisher Scientific). Where applicable, solubility tags (e.g., MBP) were removed by incubation with TEV protease for 1-2 h at 4 °C.

**In Vitro Phase Separation Assays**

Purified proteins were diluted to the indicated concentrations in phase separation buffer (typically 40 mM Tris-HCl, pH 7.4, 150 mM NaCl, 1 mM DTT). For co-condensation assays, proteins were mixed at specified ratios before dilution. Samples were incubated in 384-well low-binding microscopy plates (Greiner bio-one, 781090) for 20-30 minutes at room temperature before imaging. Droplet formation was observed using a Nikon A1 HD25 or Zeiss LSM880 confocal microscope with a 63x or 100x oil immersion objective. Images were captured with excitation/emission settings appropriate for the fluorescent tags (GFP: 488 nm; mCherry: 561 nm; BFP: 405 nm; TAMRA: 561 nm).

**Fluorescence Recovery After Photobleaching (FRAP)**

FRAP experiments were performed on confocal microscopes (Nikon A1 HD25). A defined region within a condensate was bleached using high-intensity laser light. Recovery of fluorescence into the bleached area was monitored over time by capturing images at low laser intensity at regular intervals. Fluorescence intensity within the bleached region was normalized to the pre-bleach intensity and to a reference unbleached region. Recovery curves were plotted and analyzed using ImageJ (Fiji) or the microscope's proprietary software.

**Quantification of FRAP data**FRAP data were quantified according to Carnell et al^[^[^52^](#_ENREF_52)^]^. The mobile fraction was determined from the plateau of the normalized recovery curve, and the half‑time of recovery (t₁/₂) was obtained by interpolation as the time at which fluorescence intensity reached 50% of the plateau value. All quantitative analyses were performed using at least three independent replicates, and data are presented as mean ± SD.

**Yeast transformation for heterologous expression**

The plasmids were linearized with NotI and the resulting fragments were gel-purified and transformed into the fission yeast strain LD328 (genotype his3-D1 leu1-32). Briefly, yeast cells were cultured until the OD600 reached 0.4-0.8. For each reaction, 500 μL cultured cells were collected, washed three times with sterilized water and resuspended in buffer I (240 μL of 50% PEG3350, 36 μL of 1.0 M LiAc and 50 μL of 2.0 mg/mL carrier DNA). The linearized DNA (34 μL, up to 1 μg) was added to the resuspended cells, mixed vigorously and incubated at 42°C for 40 min. The cells were collected and resuspended in 100 μL water and plated on EMM + HT (EMM medium supplemented with 45 mg/L histidine and 15 μM thiamine) plates. After incubation at 30°C for 2-3 days, individual colonies were selected on EMM + H (EMM medium supplemented with 45 mg/L histidine) plates. The cells were used for subsequent imaging analyses.

**Electrophoretic Mobility Shift Assay (EMSA)**

TAMRA-labeled double-stranded DNA probes corresponding to the wild-type or mutant *CathB3* promoter sequence were incubated with purified GBF1 protein in 1x EMSA buffer (25 mM HEPES, pH 8.0, 40 mM KCl, 5 mM MgCl₂, 1 mM EDTA, 8% glycerol, 1 mM DTT) for 1 hour at room temperature. Reactions were resolved on a pre-run 5% native polyacrylamide gel in 0.5x TBE buffer at 100 V for 60-90 minutes. Gels were scanned using a Typhoon FLA9500 imager (Cytiva) with the TAMRA channel. The probe sequence is available in Supplementary Table S1.

**Chromatin Immunoprecipitation (ChIP)-qPCR**

ChIP assays were performed using 24-h imbibed seeds (Col-0, *pGBF1::GBF1-mVenus/Col-0*, *pGBF1::GBF1-mVenus/gip1-1*) grown on filter paper over 1/2 MS sucrose-free medium. Harvested samples were cross-linked with 1% formaldehyde in PBS under vacuum for 15 min. The reaction was quenched with 0.125 M glycine. After washing, tissues were frozen in liquid nitrogen, ground to powder, and stored at −80°C. For chromatin preparation, powder was homogenized in Honda buffer (0.44 M sucrose, 1.25% Ficoll, 2.5% Dextran T40, 20 mM HEPES-KOH (pH 7.4), 10 mM MgCl₂, 0.5% Triton X-100) supplemented with 10 mM DTT and 1× protease inhibitor cocktail, filtered through Miracloth (Millipore, 475855-1RCN), and centrifuged at 4,000 ×*g* for 5 min at 4°C. The pellet was lysed in nuclear lysis buffer (50 mM Tris-HCl (pH 7.5), 10 mM EDTA, 0.5% SDS) with 2× protease inhibitor cocktail, then sonicated (15 min, 5 sec on/off, 60% amplitude). Cleared lysate was incubated overnight at 4°C with GFP-Nanoab-Magnetic Beads (LABLEAD, GNA-50-1000) pre-washed in ChIP dilution buffer (50 mM Tris-HCl (pH 7.5), 150 mM NaCl, 1 mM EDTA, 1% Triton X-100, 0.1% SDS). Beads were washed sequentially with: Buffer A (150 mM NaCl, 20 mM Tris-HCl (pH 7.5), 2 mM EDTA, 1% Triton X-100); Buffer B (500 mM NaCl, 20 mM Tris-HCl (pH 7.5), 2 mM EDTA, 1% Triton X-100); Buffer C (10 mM Tris-HCl (pH 7.5), 250 mM LiCl, 1 mM EDTA, 1% NP-40, 0.5% sodium deoxycholate); and Buffer D (10 mM Tris-HCl (pH 7.5), 1 mM EDTA, 0.1% Triton X-100). Bound complexes were eluted with elution buffer (1% SDS, 0.1 M NaHCO₃). After reversing cross-links overnight with 5 M NaCl at 65°C, DNA was purified via phenol-chloroform extraction and ethanol precipitation. Precipitated DNA was resuspended in water and analyzed by qPCR using primers specific to the *CathB3* promoter. Results were normalized to input DNA. The primer for CHIP-qPCR is shown in Supplementary Table S1.

**Transient Expression in Protoplasts and *N. benthamiana***

Arabidopsis mesophyll protoplasts were isolated from leaves of 3-4-week-old plants using cellulase and macerozyme enzymes. Protoplasts were transfected with plasmid DNA using polyethylene glycol (PEG)-mediated transformation. For transactivation assays, a reporter plasmid containing the *CathB3* promoter driving firefly luciferase (LUC) and an internal control plasmid expressing Renilla luciferase (REN) were co-transfected with effector plasmids. Luminescence was measured 16-24 hours after transfection using the Dual-Luciferase Reporter Assay System (Promega, E1910). Firefly luciferase activity was normalized to Renilla luciferase activity.

For transient expression in *N. benthamiana*, Agrobacterium tumefaciens strain GV3101 carrying the desired plasmids was grown, resuspended in infiltration buffer (10 mM MgCl₂, 10 mM MES, pH 5.6-5.7, 200 μM acetosyringone), and infiltrated into leaves of 3-4-week-old plants. Tissues were harvested 24-48 hours post-infiltration for analysis.

**Fluorescence imaging of cells and tissues**

Arabidopsis seed imaging. Arabidopsis seeds were stratified at 4°C for 3 days in the dark and then transferred to a growth chamber under light conditions. After 24 hours of imbibition, seeds were carefully dehusked under a stereomicroscope using a fine-tipped forceps. A dehusked seed was placed on a glass slide in a 10 µL droplet of liquid half-strength MS medium and gently covered with a coverslip. Imaging was performed using a Nikon AXR confocal microscope system equipped with NSPARC detectors and a ×100/1.45 NA oil immersion objective. Due to the high susceptibility of the seed fluorescence to photobleaching, a low-intensity laser was first used for preview and to identify regions of interest. Subsequently, images were acquired using a medium laser intensity for excitation and high detector gain to optimize the signal-to-noise ratio while minimizing photodamage. Autofluorescence was predominantly detected in the cytoplasm, while the GBF1-GFP signal was nuclear-localized.

For imaging of tobacco leaf epidermal cell, a small leaf disc was excised and soaked in liquid half-strength MS medium prior to imaging. The leaf disc was mounted on a glass slide and imaged immediately using a Nikon A1 HD25 confocal microscope. For imaging of yeast cell, three independent colonies were streaked onto a fresh medium plate and cultured overnight at 30°C. Before imaging, a single colony was resuspended in the appropriate liquid medium. A small droplet of the cell suspension was sprayed onto a glass slide and covered with a coverslip. Imaging was performed using a Nikon A1 HD25 confocal laser microscope and a ×100/1.45 NA oil immersion objective.

For fluorescence detection, GFP was excited at 488 nm and emission was collected at 500-550 nm; mVenus was excited at 514 nm and emission was collected at 529-570 nm.

**Fluorescence Lifetime Imaging-Förster Resonance Energy Transfer (FLIM-FRET)**

The solubility tag MBP was first removed from the GBF1-GFP fusion protein by TEV protease cleavage. The donor, GBF1-GFP (10 µM), was incubated with the acceptor, a TAMRA-labeled DNA fragment containing the *CathB3* G-box (100 nM), in 384-well microscopy plates for 30 minutes at room temperature to allow complex formation before imaging.

FLIM-FRET imaging was carried out on a Leica TCS SP8 laser-scanning confocal microscope equipped with a 100×/1.40 NA oil immersion objective and FLIM capability. The donor fluorescence (mGFP) was excited at 488 nm using a pulsed white light laser, and photon events were recorded using a time-correlated single photon counting (TCSPC) module. To accumulate sufficient photon counts for robust fitting, the selected region of interest (ROI) was repeatedly scanned 30-100 times. Photon arrival times were fitted to a double-exponential reconvolution model using the Leica LAS X FLIM FCS software, and the mean fluorescence lifetime was calculated based on intensity weighting. A decrease in the donor fluorescence lifetime indicates the occurrence of FRET and thus close proximity between GBF1 and the DNA probe. For each sample, fluorescence lifetimes were analyzed from a minimum of six distinct ROIs.

**β-Galactosidase Liquid Assay**

The *Saccharomyces cerevisiae* reporter strain EGY48 was used for all two-hybrid assays. Positive cotransformants were obtained by co-transforming the yeast strain with the pLacZi reporter plasmid (*pCathB3-LacZi*) and the pB42AD fusion plasmid (pB42AD, pB42AD-GBF1^PrLDm^, pB42AD-GBF1^FUSN^, and pB42AD-GBF1^ΔPrLD^) using a high-efficiency LiAc/PEG method. Briefly, yeast competent cells were prepared by washing overnight YPD cultures with sterile dH₂O and resuspending them in a freshly prepared One-step buffer (0.1 M LiAc, 40% PEG3350, 0.1% β-mercaptoethanol). A mixture of 2.5 µL of each plasmid and 5 µL of sheared salmon sperm DNA was added to 100 µL of competent cells. After incubation at 45°C for 30 minutes with intermittent vortexing, the transformed cells were selected on SD/-Trp-Ura solid medium and incubated at 30°C for 2-4 days. β-Galactosidase activity was quantitatively measured from liquid cultures of these positive cotransformants using the ortho-Nitrophenyl-β-galactoside (ONPG) hydrolysis assay. Single colonies were inoculated into 5 ml of SD/-Trp-Ura liquid selection medium and grown overnight at 30°C with shaking (200 rpm). The overnight culture was diluted into 8 ml of YPD medium and incubated until the cells reached mid-log phase (OD600 = 0.5-0.8). Cells were harvested, washed, and concentrated 5-fold in Z buffer. A 100 µl aliquot of the cell suspension was permeabilized by three freeze-thaw cycles using liquid nitrogen and a 37°C water bath. The enzymatic reaction was initiated by adding 700 µl of Z buffer with β-mercaptoethanol and 160 µl of ONPG substrate (4 mg/ml in Z buffer) and incubated at 30°C. The reaction was terminated by adding 400 µl of 1 M Na_2_CO_3_ upon visible yellow color development, and the elapsed time was recorded. After centrifugation to remove cell debris, the absorbance of the supernatant was measured at 420 nm. β-Galactosidase activity was calculated in Miller Units using the formula:

Activity = 1000 × OD420 / (t × V × OD600), where “t” is the reaction time (min), *V* is the volume of culture assayed (0.1 ml × concentration factor), and *OD600* is the optical density of the original culture. All assays were performed in triplicate.

**Yeast One-Hybrid Assay for transcriptional activation of GAL4**

The constructed plasmids (pBKT7, pBKT7-GBF1, pBKT7- GBF1^PrLDm^, pBKT7- GBF1^FUSN^, pBKT7-GBF1^ΔPrLD^) was individually transformed into the *Saccharomyces cerevisiae* reporter strain AH109, which contains integrated GAL4-responsive reporter genes (*HIS3*, *ADE2*), using the high-efficiency LiAc/PEG method as previously described. Positive transformants were selected on SD/-Trp solid medium. For transcriptional activation analysis, three independent colonies of each transformation were spotted onto SD/-Trp control plates and SD/-Trp/-His/-Ade triple-dropout selection plates. The plates were incubated at 30 °C for 3-5 days, and growth was documented photographically. Activation of the reporter genes was assessed based on colony growth under selective conditions.

**Yeast Two-Hybrid Assay**

The constructed plasmids (pGADT7, pGBKT7, pGADT7-GBF1, pGBKT7-GIP1) were co-transformed into yeast strain AH109, using the high-efficiency LiAc/PEG method as previously described. Transformants were selected on synthetic dropout (SD) medium lacking Leu and Trp (-LW). Protein interactions were assessed by growth on SD medium lacking Leu, Trp, His, and Ade (-LWAH).

**Turbidity measurements**

Purified MBP-GBF1 was treated with TEV protease to remove the MBP tag. The cleaved GBF1 protein was then diluted to a final concentration of 5 μM in phase separation buffer (40 mM Tris-HCl, pH 7.4, 150 mM NaCl) and incubated with increasing concentrations of GIP1 (0, 1, 3, and 5 μM). The mixtures were transferred to a flat-bottom 96-well plate (Corning, 3364), and turbidity was measured at 600 nm using a Varioskan Flash microplate reader (Thermo Scientific). All measurements were performed in triplicate.

**Redox Treatment and Electrophoretic Analysis of GIP1**

Wild-type GIP1 was incubated in either a reducing buffer (5 mM DTT, 500 mM NaCl, 20 mM Tris-HCl, pH 7.4) or an oxidizing buffer (3 mM H₂O₂, 500 mM NaCl, 20 mM Tris-HCl, pH 7.4) for 1 hour at room temperature. To remove DTT or H₂O₂ while maintaining the protein in its reduced or oxidized state, the samples were subjected to buffer exchange using centrifugal concentrators, with three to four washes in a storage buffer (500 mM NaCl, 20 mM Tris-HCl, pH 7.4). The redox states of the treated proteins were then analyzed by non-reducing 8% SDS-PAGE. Samples for non-reducing conditions were prepared in loading buffer without β-mercaptoethanol, while a control sample was prepared in loading buffer containing β-mercaptoethanol to represent the fully reduced form.

**Statistical Analysis**

Data are presented as mean ± standard deviation (SD) or standard error of the mean (SEM) from at least three independent biological replicates, as indicated in the figure legends. For comparisons between two groups, statistical significance was determined using unpaired two-tailed Student’s t-test, with significance levels indicated by asterisks (***P < 0.001, **P < 0.01, *P < 0.05). For comparisons involving more than two groups, one-way ANOVA followed by Tukey’s post-hoc test was used, and groups with different letters indicate significant differences (P < 0.05). Statistical analyses were performed using IBM SPSS Statistics or GraphPad Prism software.
